# Assessing urogenital schistosomiasis and female genital schistosomiasis (FGS) among adolescents in Anaocha, Anambra State, Nigeria: implications for ongoing control efforts

**DOI:** 10.1186/s12889-024-18378-0

**Published:** 2024-04-02

**Authors:** Ogechukwu B. Aribodor, Nwadiuto O. Azugo, Eunice C. Jacob, Uche C. Ngenegbo, Nnaemeka D. Onwusulu, Ifeanyi Obika, Emmanuel M. Obikwelu, Obiageli J. Nebe

**Affiliations:** 1 Department of Zoology, Nnamdi Azikiwe University, Awka, Nigeria; 2 Social Innovation in Health Initiative (SIHI) Hub, Nnamdi Azikiwe University, Awka, Nigeria; 3 Department of Parasitology and Entomology, Nnamdi Azikiwe University, Awka, Nigeria; 4 Department of Obstetrics and Gynaecology, Nnamdi Azikiwe University, Awka, Nigeria; 5 Neglected Tropical Diseases Unit, Anambra State Ministry of Health, Awka, Nigeria; 6 Neglected Tropical Diseases Division, Federal Ministry of Health, Abuja, Nigeria

**Keywords:** Female genital schistosomiasis, Urogenital schistosomiasis, Praziquantel preventive chemotherapy, Neglected tropical diseases, Adolescents, Colposcopy examination, FGS lesions, Sexual and reproductive health services, Behavior change communication

## Abstract

**Background:**

Urogenital schistosomiasis (UgS) remains a persistent health challenge among adolescents in Anambra State, Nigeria, despite ongoing control efforts. Mass praziquantel treatment programs, initiated in 2013, primarily target primary school-aged children (5–14 years old), leaving adolescents (10–19 years old) enrolled in secondary schools vulnerable to urogenital schistosomiaisis. Additionally, the extent of female genital schistosomiasis (FGS), a neglected gynaecological manifestation of UgS remains unclear.

**Methodology:**

To address these gaps, a cross-sectional study was conducted in Anaocha Local Government Area from February to May 2023. Four hundred and seventy consenting adolescents aged 10–19 years were enrolled. Urinalysis including urine filtration was employed to confirm haematuria and detect urogenital schistosomiasis (UGS) among the participants. For females with heavy infections (≥ 50 eggs/10 ml urine), a gynaecologist performed colposcopy examinations, complemented by acetic acid and Lugol’s iodine staining to assess for female genital schistosomiasis (FGS) lesions or other related reproductive health conditions. Socio-demographic data, including information on potential risk factors, were systematically collected using the Kobo ToolBox software, following gender-sensitive data collection guidelines. Data were analysed using SPSS version 25, incorporating descriptive statistics, multinomial logistic regression, odds ratios, and significance testing.

**Results:**

Among the 470 adolescents (52.8% females, 47.2% males) examined, an overall UgS prevalence of 14.5% was observed, with an average of 5.25 eggs per 10 ml of urine. Females had a slightly higher prevalence (16.1%), and 7.5% had heavy infections. Although gender differences in infection rates were not statistically significant, males had slightly higher odds of infection (OR: 1.332; 95% CI: 0.791–2.244; p-value: 0.280). Adolescents aged 10–14 had the highest prevalence, with significantly increased odds of infection (OR: 1.720; 95% CI: 1.012–2.923; p-value: 0.045). Colposcopy examinations of females with heavy infections revealed FGS lesions and co-infections with *Trichomonas vaginalis.* Haematuria, though prevalent (24.6%), was not the sole indicator, as those without it faced significantly higher odds of infection (OR: 2.924; 95% CI: 1.731–4.941; p-value: 0.000). Dysuria and genital itching/burning sensation were other UgS and FGS associated symptoms. Direct water contact was associated with higher infection odds (OR: 2.601; 95% CI: 1.007–6.716; p-value: 0.048). Various risk factors were associated with UgS.

**Conclusion:**

The study highlights the need for a comprehensive Urogenital Schistosomiasis (UGS) control strategy that includes secondary school adolescents, emphasizes risk factor management, promotes safe water practices, and raises awareness about UGS and Female Genital Schistosomiasis (FGS) among adolescents, thus improving control efforts and mitigating this health challenge in the region.

**Supplementary Information:**

The online version contains supplementary material available at 10.1186/s12889-024-18378-0.

## Background

### Background and significance

Urogenital schistosomiasis (UgS), caused by *Schistosoma haematobium*, is a significant public health challenge, especially in sub-Saharan Africa [1]. This parasitic disease spreads through contact with fresh water contaminated by snails carrying infective larvae known as cercariae and results in various health problems [2].

### Epidemiological status in Nigeria

In Nigeria, UgS is endemic across all 36 states [3], with approximately 101 million people at risk, primarily school-aged children and adolescents [4]. Nigeria shoulders a substantial burden of UgS within sub-Saharan Africa [4, 5]. However, there is a scarcity of reliable, comprehensive reports on the disease’s epidemiological status in many regions of the country [6]. Only in 2015 did Nigeria’s Federal Ministry of Health release the first official epidemiological data, revealing a prevalence of 9.5% [6, 7].

The transmission of urogenital schistosomiasis (UgS) is facilitated by socio-cultural and biological factors. Engaging in activities like washing, fishing, and recreation in freshwater environments where infected snails are present is a significant risk factor for UgS transmission due to the presence of snails infected with *S. haematobium* in aquatic habitats [3]. The snails are essential for the parasite to develop, multiply, and infect new hosts via water contact. The parasite population in snail hosts increases through asexual reproduction, where a single miracidium can infect a snail and produce hundreds or thousands of cercariae that infect new hosts [8]. Environmental factors, including temperature, altitude, rainfall, and land use cover, significantly influence disease distribution. For instance, temperature and altitude impact intermediate host snail survival and reproduction, while rainfall creates temporary snail habitats [9]. Additionally, water level and velocity, rather than just the season, determine snail host activity. Socio-demographic factors such as age [6, 10, 11] and gender [10, 12] may also affect the prevalence of infection. School-aged children and young adults, for instance, are at a higher risk of infection due to their frequent water contact activities [13]. Other sociocultural and behavioural factors such as cultural barriers [14, 15], misinformation [16], misconceptions [17, 18], and poor health-seeking behaviours [19] perpetuate transmission. Socioeconomic disparities in low-and-middle-income countries contribute to significant public health challenges regarding the transmission of the disease [20]. Also, the internal migration of displaced individuals due to insurgencies, insecurity, and flooding may contribute to the spread of the disease [21].

The prevalence of UgS in Nigeria varies from region to region, with some areas having much higher prevalence [21–24] than others [10, 25–27].

### Clinical manifestations of urogenital schistosomiasis

Schistosomiasis has three stages: cercarial dermatitis, acute schistosomiasis, and chronic schistosomiasis [28]. Cercarial dermatitis results from schistosome cercariae penetrating human skin, causing an allergic inflammatory skin lesion [29]. Acute schistosomiasis, occurring 3–8 weeks post-infection, presents with symptoms like prolonged fever, weakness, vomiting, nausea, diarrhoea, malaise, and rapid weight loss [30]. Chronic schistosomiasis arises due to granulomatous inflammation from parasite egg accumulation in various tissues [30]. Urogenital schistosomiasis (UgS) manifests as a chronic immune-mediated disease affecting the urinary and genital tracts. It is characterized by symptoms such as haematuria (blood in urine) and persistent inflammation caused by the presence of eggs trapped in the tissues of pelvic organs, including the urinary bladder, lower uterus, cervix, vagina, prostate gland, and seminal vesicles. This inflammation leads to tissue destruction, fibrosis, granuloma formation, and the development of fibrotic nodules commonly referred to as sandy patches [31]. The severity and frequency of UgS complications depend on infection intensity and duration [31, 32].

### Gender specific manifestation of urogenital schistosomiasis

The term “urogenital schistosomiasis” encompasses urinary and genital manifestations [33]. In women, it manifests as female genital schistosomiasis (FGS), marked by symptoms like abnormal vaginal discharge, abdominal and pelvic pain, discomfort during sexual intercourse, bleeding after sexual activity, and cervical epithelial changes [17, 33–35]. Cervicovaginal manifestations of FGS manifest as visible lesions, including grainy sandy patches, homogenous yellow sandy patches, rubbery papules, or abnormal blood vessels [36]. In men, it is called male genital schistosomiasis (MGS), leading to pain and discomfort during pelvic activities, abnormal ejaculation (including haemospermia and leukocytospermia), and genital swelling [33, 37, 38].

### Neglected impact of female genital schistosomiasis

It is interesting to note that FGS, a neglected gynaecological manifestation of UgS affecting 56 million girls globally [39], is often overlooked even in the health curriculum [18] and is not separately assessed in the global disease burden studies [39]. The FGS can result in various complications, including genital itching, pain, bleeding, and an increased susceptibility to human papillomavirus (HPV) infections [40]. These consequences extend beyond physical health, affecting mental well-being and reproductive health [10, 41]. Furthermore, FGS is intricately linked to the spread of HIV/AIDS, making it a critical area of concern for public health efforts [10, 41].

### Diagnostic challenges

Diagnosing urogenital schistosomiasis (UgS) in resource-limited areas presents significant challenges. Common diagnostic methods, such as urinalysis strips to detect blood in urine, often yield false positives. Microscopic examination of urine samples, a crucial diagnostic step for UgS, and centrifugation, required for this process, rely on access to electricity, which may be unavailable. Additionally, urine filtration, another diagnostic approach, can be expensive [42]. Furthermore, colposcopy, the method used to diagnose female genital schistosomiasis (FGS), faces difficulties due to the scarcity of equipment and its invasive nature [43, 44].

This reliance on these methods underscores the pressing need to address equipment and procedural limitations. It is of utmost importance to develop and implement reliable diagnostic tools to enhance the effectiveness of interventions and control measures [42].

### Gaps in treatment programmes

Control programs have traditionally prioritized deworming primary school children, inadvertently neglecting adolescents, enrolled in secondary schools, and other vulnerable demographic groups [10, 45]. In 2013, Anambra State launched mass praziquantel administration, primarily targeting school-aged children aged 6 to 12 [10, 46, 47]. This approach leaves adolescents, such as a 16-year-old girl residing near Agulu Lake in Anaocha Local Government Area, susceptible to UgS and FGS. Despite her ineligibility for praziquantel treatment meant for younger children, she faces daily exposure to schistosome cercariae while performing household chores and assisting on the family farm.

These untreated adolescents and vulnerable individuals can serve as reservoir hosts, perpetuating the parasite’s transmission in the environment. Additionally, there is a potential risk of them becoming sources of infection if they migrate to non-endemic areas [48].

Furthermore, the limited evidence on the impact of ongoing Mass Drug Administration (MDA) programs in Anambra State underscores the need for surveillance, monitoring, and evaluation efforts. Misclassifying communities as non-endemic can lead to restricted access to preventive chemotherapy (PC) programs [10]. This knowledge gap not only hinders our understanding of the initiatives’ effectiveness but may also inadvertently deprive certain populations and communities of essential treatments.

### Research Objective and significance

This study is of utmost significance for the ongoing control efforts against urogenital schistosomiasis (UgS) and female genital schistosomiasis (FGS) among adolescents in Anaocha, Anambra State, Nigeria. By assessing the prevalence and risk factors of UgS and FGS in this specific demographic, the research provides essential data to inform targeted interventions. These interventions are crucial not only for improving the reproductive health and overall well-being of adolescents but also for strengthening control measures in the region. The study’s findings underscore the imperative need for tailored surveillance, monitoring, and evaluation, especially to assess the impact of the Mass Drug Administration (MDA) programs that have been ongoing. Furthermore, this research contributes to the global effort to eliminate neglected tropical diseases, promoting health equity, and advancing gender equality.

## Methods

### Study area

**Fig. 1:**
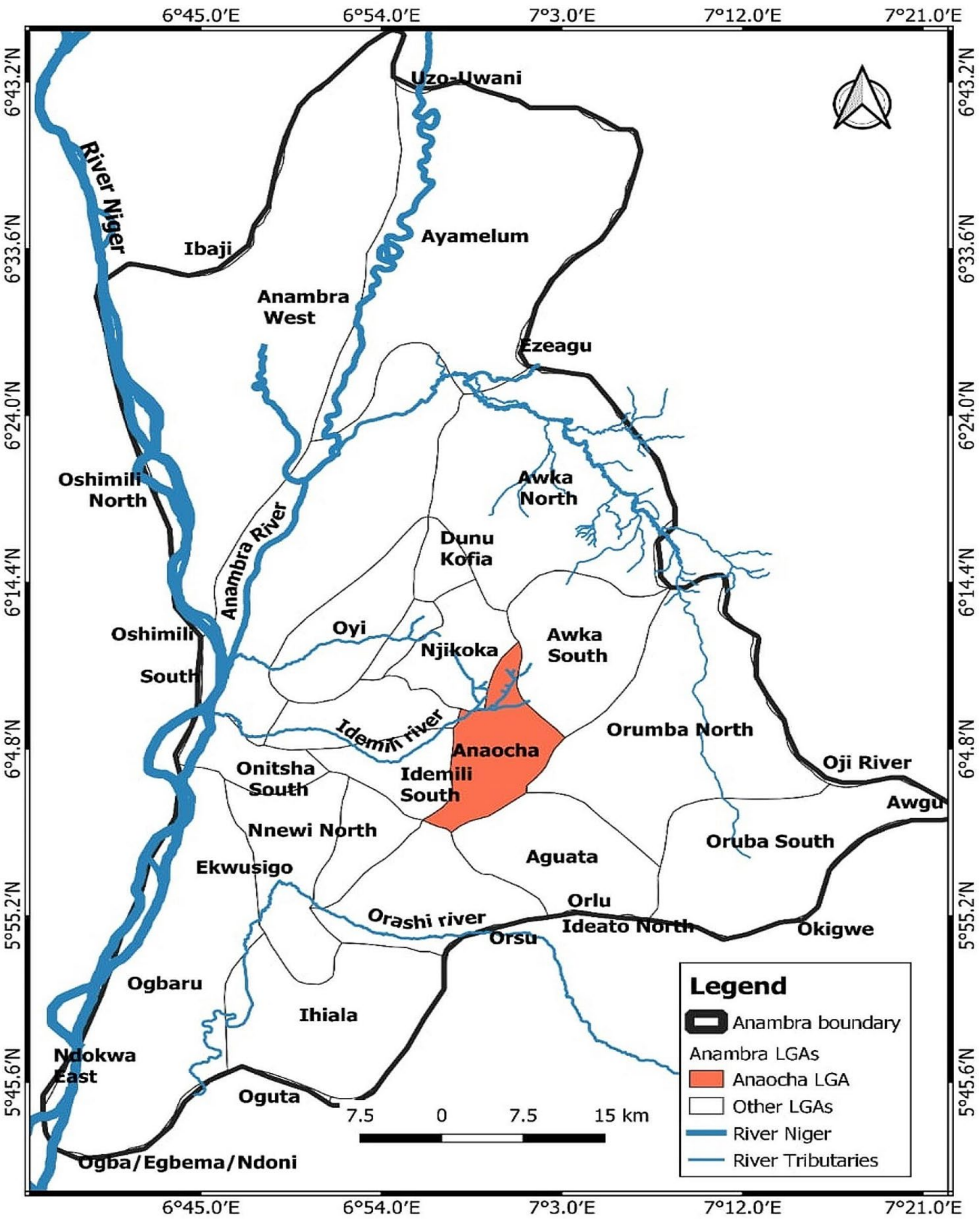
Map of Anambra State Showing the Selected Local Government Area for the Study (Source: Geography Information System Laboratory, Department of Estate Survey and Geoinformatics, Nnamdi Azikiwe University, 2023)

The study was conducted in Anaocha Local Government Area of Anambra State, Nigeria (Fig. 1), which is known for its diverse watery ecosystems, including rivers, ponds, irrigated farmland, and burrow pits [49]. These ecosystems provide favourable habitats for aquatic snails that serve as intermediate hosts for *Schistosoma haematobium*. Anaocha LGA is characterized by uplands, lowlands, stream channels, and gullies, with the Anambra River and its tributaries flowing through the area [50]. The region experiences a tropical climate with distinct wet and dry seasons, high humidity during the wet season, and relatively constant temperatures throughout the year [51]. Water levels in rivers and lakes fluctuate with the seasons, rising during the wet season and falling during the dry season [52]. The snail hosts of *S. haematobium* are more active during the rainy season when they lay eggs in the water, while they go dormant in the mud at the bottom of water bodies during the dry season. This behaviour is primarily influenced by water levels and velocity within their habitat, rather than being solely dependent on the season. These snails can withstand high temperatures, making the area ideal for their survival [53].

Anaocha LGA has a population of approximately 418,360 people across ten communities, with Adazi-Nnukwu and Agulu chosen for the study due to their proximity to Agulu Lake and other water bodies [54, 55]. Adazi-Nnukwu is a semi-urban community primarily engaged in farming, with two rivers within the community. Agulu is a semi-urban town known for Agulu Lake, the largest lake in Anambra State, and various other rivers and ponds. These communities are situated within a network of neighbouring towns and villages in the region.

### Study design

The study was a community-based cross-sectional study conducted from February 2023 to May 2023, and two communities, Adazi-Nnukwu and Agulu, within Anaocha Local Government Area were chosen for the study using purposive sampling due to their proximity to Agulu Lake and other freshwater bodies. Previous studies have also implicated these communities as endemic areas for urinary schistosomiasis [27, 49, 56].

### Study population and sample size determination

**Fig. 2:**
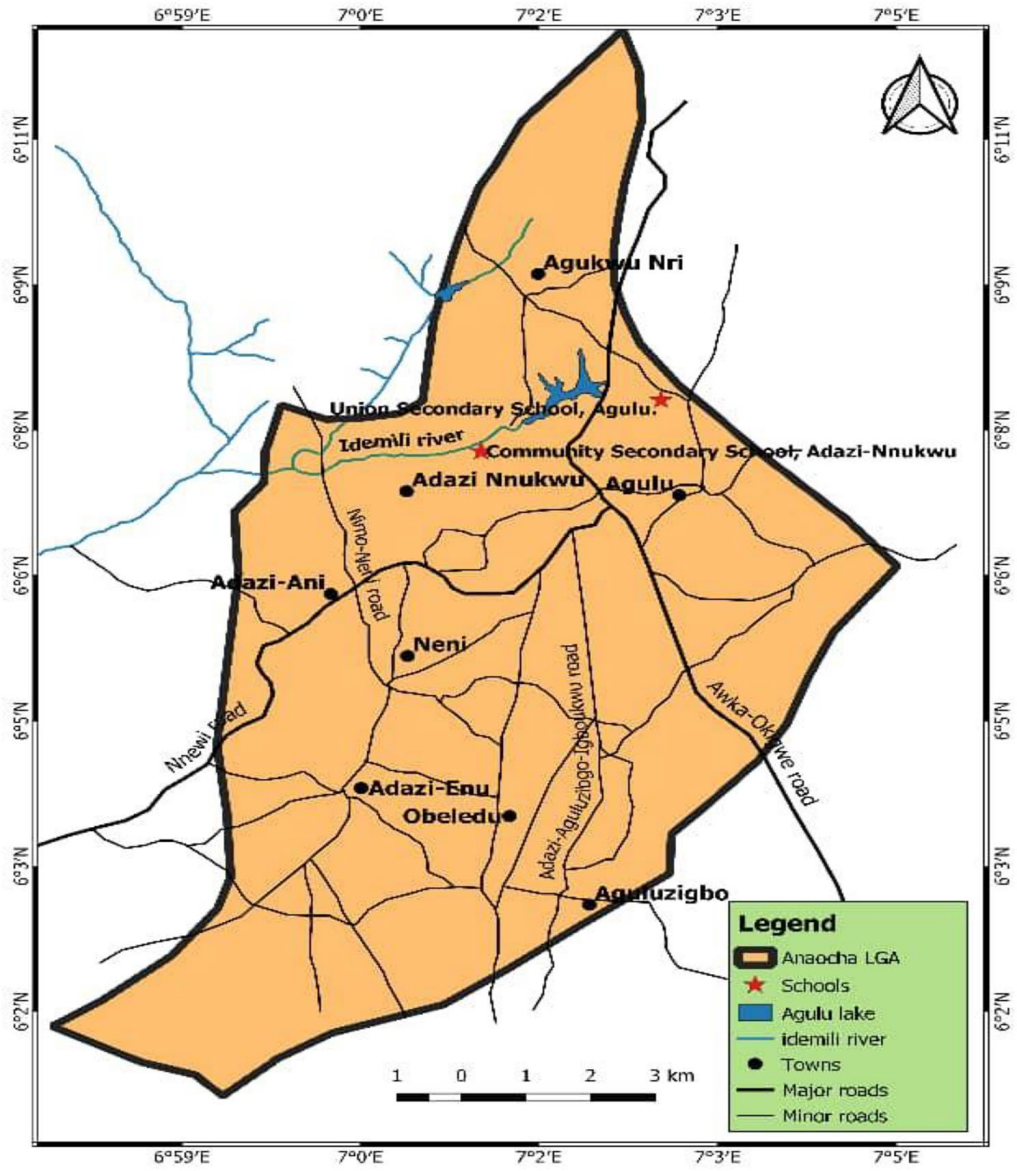
Map of Anaocha Local Government Area Showing the Locations Selected for the Study (Source: Geography Information System Laboratory, Department of Estate Survey and Geoinformatics, Nnamdi Azikiwe University, 2023)

The study population consisted of 470 consenting and assenting adolescents aged between 10 and 19 years who were enrolled in Community Secondary School, Adazi-Nnukwu, and Union Secondary School, Agulu (Fig. 2).

The sample size of 470 was determined using the formula [57]: $n =\frac{N}{1+N{\left(e\right)}^{2}}$

Where:

n = sample size.

N = population size.

e = level of precision or sampling error which is taken to be 5% here.

Anambra State Population of Students as obtained from the Post Primary School Service Commission of Anambra State, *N* = 112, 255.

e = 0.05. $$ n =\frac{112,255}{1+112,255{\left(0.05\right)}^{2}} = 398.6$$

To account for a 75% response rate [58] and a 25% non-response rate, an additional 18% of the sample size ( $\frac{18}{100}\times 398.6$ = 71.75+398.6 = 470.4) was calculated and included, resulting in a final sample size of 470 participants to optimize precision and minimize potential withdrawals. A stratified sampling technique was employed [59], with participants selected randomly from five strata representing different classes, ranging from junior secondary class 1 (JS 1) to senior secondary class 2 (SS2).

Inclusion criteria mandated that the participants be aged between 10 and 19, enrolled in one of the designated public secondary schools, and provided written consent for their involvement. Females menstruating at the time were excluded to prevent the erroneous detection of hematuria in urine samples.

### Data collection

Data for this study were collected through a combination of qualitative and quantitative methods to ensure a comprehensive understanding of the research area and its participants.

#### Qualitative data collection

Structured questionnaires were administered to participants through one-on-one interviews conducted in English and Igbo language only, with the latter, translated back to English language by the researchers who are fluent in Igbo language adhering to the guidelines set by the COUNTDOWN consortium for neglected tropical diseases [60]. These questionnaires were designed to gather qualitative data related to socio-demographics and various risk factors associated with urogenital schistosomiasis and female genital schistosomiasis.

Participant observation, conducted within the framework of ethnography, allowed for an insider’s perspective on the cultures and people of the study area. In-depth ethnographic interviews and the careful examination of contextual intricacies were employed to gain insights into social life among the participants [34].

#### Quantitative data collection

The structured questionnaires also included quantitative components, incorporating closed-ended questions with predefined response options. This approach facilitated the collection of quantitative data, such as demographic information and numerical risk factor assessments.

Data collection was facilitated using the KOBO ToolBox, an open-source tool for data collection and offline descriptive analysis [61]. This digital platform ensured efficient data management and analysis while maintaining a systematic approach. Privacy and gender sensitivity were prioritized during questionnaire administration, and measures were implemented to exclude menstruating female participants to prevent erroneous detection of hematuria in urine samples.

### Sample collection

Participants were provided with sterile containers to collect fresh urine samples. Urine collection took place during a specific period known for maximum schistosome egg excretion, between 10:00 and 14:00. Strict supervision was maintained to guarantee proper urine sample collection, which was then stored on ice packs and transported to the Laboratory of the Department of Zoology, Nnamdi Azikiwe University, Awka, Anambra State, Nigeria for parasitological analysis.

### Parasitological examination

The examination of samples involved a comprehensive approach, incorporating both macroscopic and microscopic methods. The macroscopic examination included the assessment of microhaematuria, urine colour, and consistency. A reagent strip (Meditest Combi 9 test strip, manufactured by Macherey-Nagel GmbH and Company, Germany) was immersed in urine for colour change observation for haematuria in the macroscopic examination. Additionally, microscopic analysis was conducted after filtration to identify terminal-spined *Schistosoma haematobium* eggs. For each urine sample, the urine filtration technique was used, and two slides were also prepared for the microscopic examination. To prepare each sample, a sterile syringe, a filtration tube with a 13 mm diameter O-ring rubber seal, and a polycarbonate membrane filter with a diameter of 13 mm and a pore size of 20.0 microns, manufactured by Sterlitech Laboratories, United States of America, were used to capture *S. haematobium* eggs. The transparent polycarbonate membrane filter was delicately handled with forceps, ensuring that the shiny side was facing up, and then carefully inserted into the cone-shaped filtration kit, and covered properly. Approximately 10 ml of urine was extracted from the sample container using the syringe, which was securely attached to the filtration kit. By flushing the urine in the syringe through the filtration kit containing the membrane filter into a sterile beaker, the urine was manually filtered using the syringe and the polycarbonate membrane filter. This filtration process captured any eggs present in the urine. Subsequently, the syringe was detached. The membrane filter, holding the captured eggs, was gently placed on a well-labelled, sterile microscope slide, with codes peculiar to each of the participants which corresponded with the information on their sterile sample containers and their questionnaires. This was done to avoid mix-ups that could lead to misdiagnosis. This procedure was repeated to prepare another slide. Both slides were then mounted onto a microscope’s stage and viewed to identify the terminal-spined *Schistosoma haematobium* eggs. The eggs were identified by their distinctive terminal spine under microscopic examination, using ×10 and ×40 magnifications [62].

### Medical examination

Female participants with a heavy intensity of *S. haematobium* eggs underwent colposcopy conducted by a gynecologist, with the affiliation as stated in the co-authors’ address section at the local general hospital. This examination aimed to assess egg lodgment in the vaginal canal, identify clinical manifestations of female genital schistosomiasis (FGS), and use the WHO FGS Pocket Atlas [63] for image analysis. After examining for FGS lesions, acetic acid was applied to the cervicovaginal region to identify precancerous lesions, and Lugol’s iodine solution was used to stain abnormal cells in the squamous epithelia, to identify other reproductive health conditions. Stringent ethical considerations were followed, including obtaining informed consent from participants and authorization from parents or guardians.

### Quality control

Quality control measures were meticulously implemented throughout the study. Field assistants received training in questionnaire administration and sample collection. Researchers ensured questionnaire completeness and adherence to recommended timeframes for urine sample collection. All slides were initially examined by skilled laboratory technologists with expertise in parasitology. To ensure the accuracy and reliability of the results, a random 10% of the slides were re-examined by one of the laboratory technologists, who was blinded to the initial results and independently examined a subset of the slides. This double-checking process was implemented to verify the original egg count, maintain the integrity of the results, and minimize the risk of false positives. The results from both technologists’ examinations were then compared for consistency.

### Data analysis

The data collected from the questionnaires underwent thorough checks for accuracy and completeness using Kobo Toolbox. Subsequently, the data was exported to Microsoft Excel and coded before being analyzed using the Statistical Package for the Social Sciences (SPSS) version 25. The analysis included descriptive statistics, categorization of infection intensity, and multinomial logistic regression to assess the association between risk factors and urogenital schistosomiasis, with statistical significance determined by *p*-values below 0.05. Infection intensity was categorized as “light” when the egg count ranged from 1 to 49 eggs per 10 ml of urine, and as “heavy” when the count exceeded 49 eggs [64]. Mean intensity was calculated using the Analysis of Variance (ANOVA) test.

## Results

### Socio-demographic assessments

A total of 470 fresh urine samples were collected from consenting and assenting adolescents enrolled in the study. The adolescents examined consisted of 52.8% (248/470) females and 47.2% (222/470) males. The mean age was 14.4 (± 2.501) years. 51.9% (244/470) were within the age group of 10–14 years, while 48.1% (226/470) were within the age group of 15–19 years. Adolescents in class JS 1 had the highest population at 31.3% (147/470), while those in JS 3 had the lowest population at 10.4% (49/470). The 39.1% (184/470) of the adolescents examined were from Adazi Nnukwu, while 60.9% (286/470) were from Agulu (Table 1).

**Table 1:** Socio-demographic characteristics of adolescents in Anaocha LGA, Anambra State, Nigeria (*N* = 470)

Variable	Category	Frequency (n)	Percentage (%)
Gender	Male	222	47.2
	Female	248	52.8
Age (years)	10–14	244	51.9
	15–19	226	48.1
Class	JS 1	147	31.3
	JS 2	82	17.4
	JS 3	49	10.4
	SS 1	92	19.6
	SS 2	100	21.3
Location	Agulu	286	60.9
	Adazi Nnukwu	184	39.1

### Prevalence of urogenital schistosomiasis and association with socio-demographic factors

**Fig. 3:**
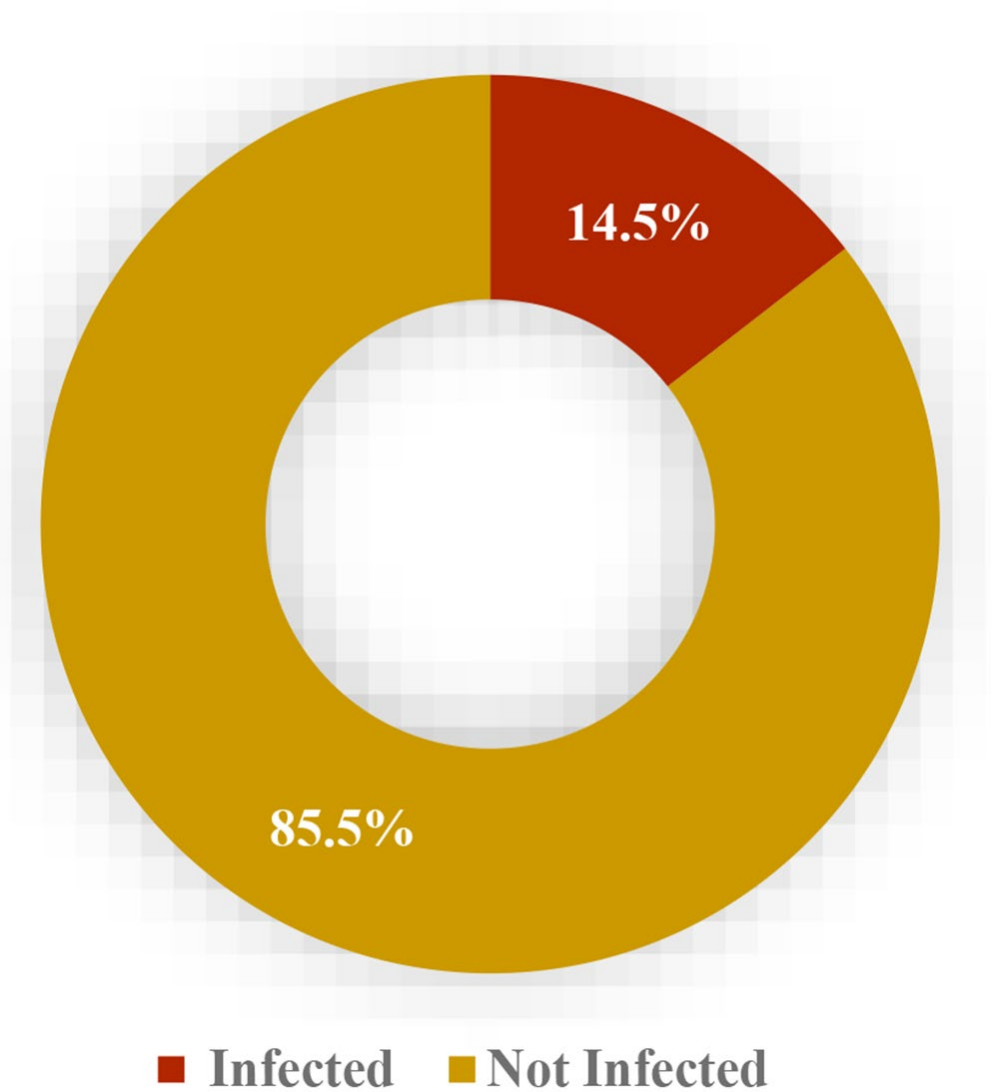
The overall prevalence of urogenital schistosomiasis among adolescents in Anaocha, Anambra State, Southeastern Nigeria

Results showed that an overall prevalence (14.5%; 68/470) of urogenital schistosomiasis among the study population as seen in Fig. 3.

**Fig. 4:**
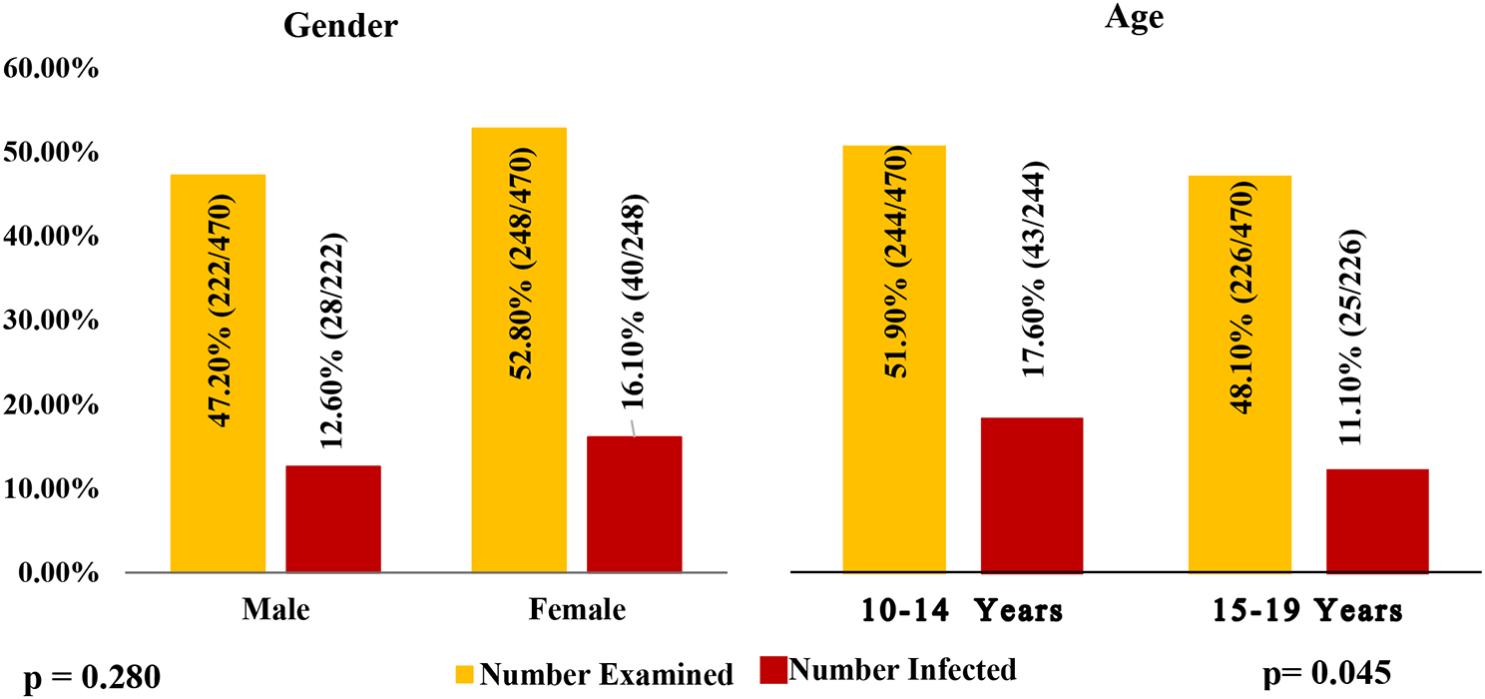
Overall prevalence of urogenital schistosomiasis with respect to gender and age

The adolescents examined were 52.8% (248/470) females and 47.2% (222/470) males. Although the females had a prevalence of infection (16.1%) that was higher than the prevalence (12.6%) among males, the difference was not statistically significant (*p* > 0.05) (Fig. 4). The results (Fig. 4) also showed that the age group 10–14 years had the highest prevalence of 17.6% (43/244).

**Fig. 5:**
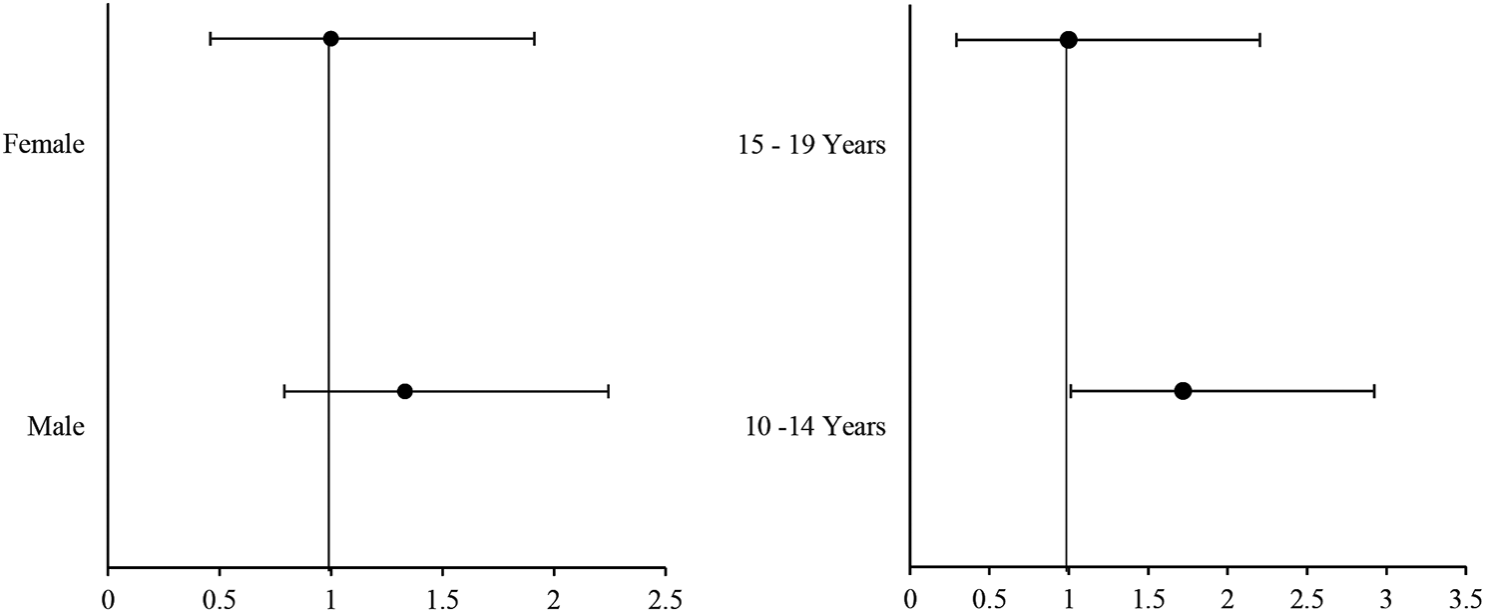
Forest plot showing the odds ratios for the association between gender, age and urogenital schistosomiasis among adolescents studied

The regression analysis can be found in Fig. 5, which showed that the males had higher odds of infection than females (OR: 1.332; 95% CI: 0.791–2.244). The regression analysis also showed that the odds of urogenital schistosomiasis for the age group below 15 years (10–14 years) were 1.720 times higher compared to the age group above 15 years, with a 95% confidence interval of 1.012–2.923 (Fig. 5).

There was a significant association between the age of the adolescents in Anaocha LGA and urogenital schistosomiasis (*p* < 0.05). Those in class JS 1 had the highest prevalence. Adazi Nnukwu also recorded the highest prevalence of infected adolescents (Table 2).

**Table 2:** Multinomial logistic analysis showing correlation between urogenital schistosomiasis and socio-demographic factors

Gender	NE	NI (%)	OR (95% CI)	p-value
Male	222	28 (12.6)	1.332 (0.791–2.244)	0.280^NS^
Female^*^	248	80 (34.8)	1.00	
**Age (years)**				
10–14	244	43 (17.6)	1.720 (1.012–2.923)	0.045^S^
15–19^*^	226	25 (11.1)	1.00	
**Class**				
JS 1	147	36 (24.5)	0.420 (0.207–0.856)	0.017^*S*^
JS 2	82	14 (17.1)	0.662 (0.288–1.524)	0.333^*NS*^
JS 3	49	0 (0.0)	X	X
SS 1	92	6 (6.5)	1.955 (0.702–5.442)	0.200^*NS*^
SS 2	100	12 (12.0)	1.000	
**Location**				
Adazi-Nnukwu	286	38 (13.3)	1.271 (0.756–2.137)	0.365^*NS*^
Agulu^*^	184	30 (16.3)	1.000	
**Total**	**443**	**68 (14.5)**		

NE = Number Examined. NI = Number Infected. OR = Odds Ratio. CI = Confidence Interval. a = Reference Category. S = Significant difference *p* < 0.05. NS = No Significant difference *p* > 0.05. X = Undefined values due to 0.0% prevalence of infection. (Multinomial Logistic Regression)

#### Intensity of urogenital schistosomiasis among the study population

Results showed that of the 68 adolescents that were infected, the mean intensity of infection among the study population ranged from 1 to 55 eggs/10 ml of urine with the overall arithmetic mean of 5.25 (± 11.330) eggs/10 ml of urine at 95% CI (2.508–7.992) (Table 3). Light-intensity infections of *S. haematobium* were observed in 95.6% (65/68) of the adolescents studied intensity, while 4.4% (3/68) had heavy intensity (≥ 50 eggs/10 ml). Table 3 showed that only light intensity of urogenital schistosomiasis (< 50 eggs/10 ml) was seen males at 100% (28/28). Females had both light intensity of UgS at 92.5% (37/68) and heavy intensity of UgS at 7.5% (3/40) at 95% CI (2.355–11.645). This was not statistically significant (*p* = 0.129).

Conversely, among adolescents aged 10–14 years, exclusively light intensity of urogenital schistosomiasis (< 50 eggs/10 ml) was detected, accounting for 100% (43/43) of cases. In contrast, older adolescents aged 15–19 years exhibited a broader spectrum of urogenital schistosomiasis intensity. Specifically, 88% (22/65) were found to have light intensity, while 12.0% (3/25) exhibited heavy intensity. This observed variation between the two age groups was found to be statistically significant (*p* = 0.005), with a 95% CI (2.5076–7.992).

**Table 3:** Mean Intensity of urogenital schistosomiasis among the infected study population with respect to gender and age

Gender	NI (%)	Mean Interval (x̄ ± σ) eggs/10 ml	Light Intensity (%)	Heavy Intensity (%)	95% CI	P-value
Male	28 (41.2)	2.75 ± 1.691	28 (100.0)	0(0.0)	2.094–3.406	0.129^*NS*^
Female	40 (58.8)	7.0 ± 14.523	37(92.5)	3(7.5)	2.355**–**11.645	
Total	**68 (100)**	**5.25 ± 11.330**	**65 (95.6)**	**3 (4.4)**	**2.508–7.992**	
**Age (years)**						
10–14	43 (63.2)	2.37 ± 2.127	43(100.0)	0 (0.0)	1.717–3.027	0.005^*S*^
15–19	25 (36.8)	10.20 ± 17.609	22(88.0)	3(12.0)	2.931–17.469	
Total	**68 (100)**	**5.25 ± 11.330**	**65 (95.6)**	**3 (4.4)**	**2.5076–7.992**	

NI = Number Infected. CI = Confidence Interval. S = Significant difference *p* < 0.05. NS = No Significant difference *p* > 0.05 (ANOVA)

### Clinical manifestations

#### I. confirmation of female genital schistosomiasis in affected adolescent girls through colposcopy

**Fig. 6:**
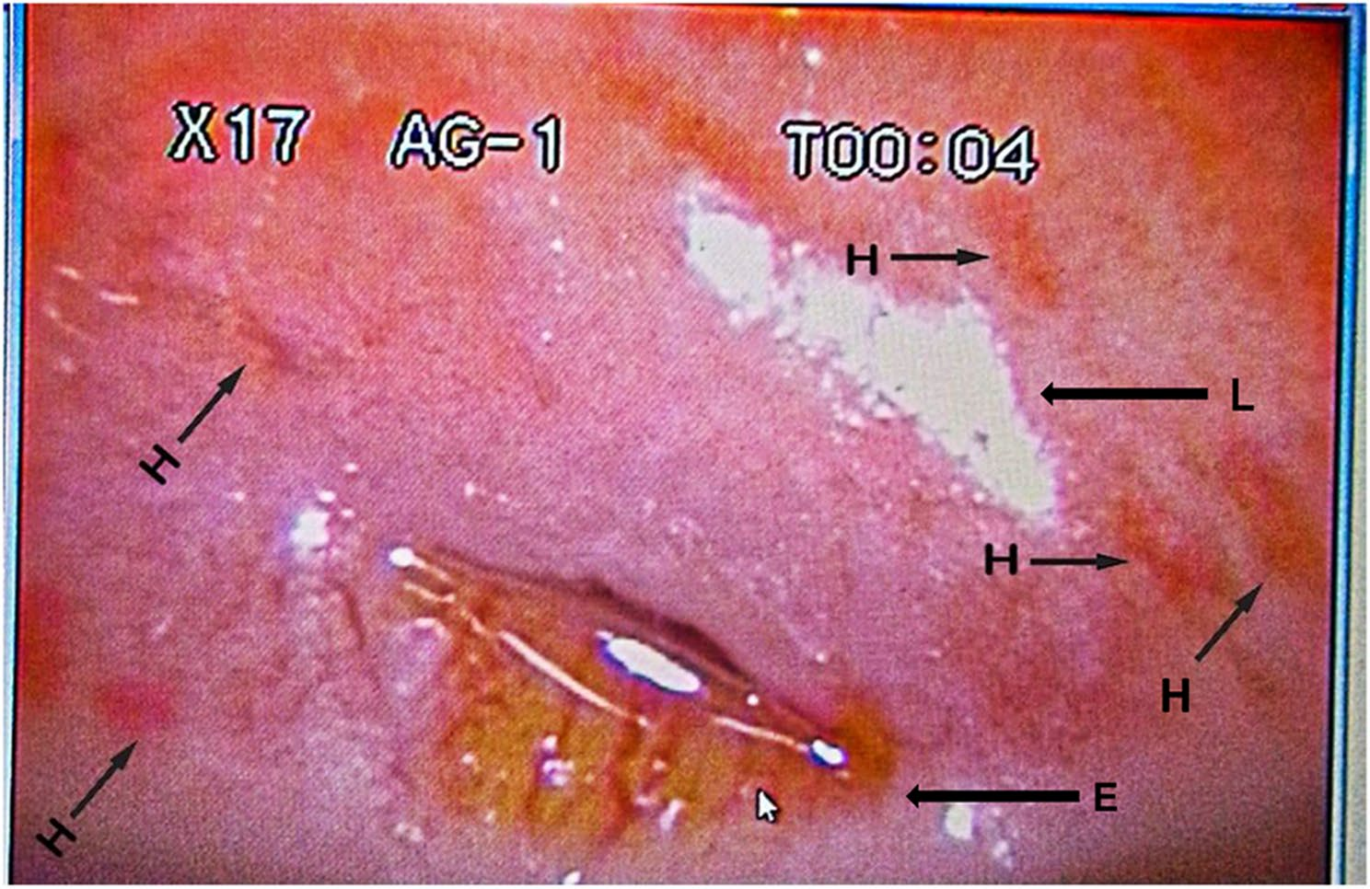
Colpophotograph of homogenous yellow sandy patches (H) and light artefact (L), surrounding the external Os (E) of the cervix

**Fig. 7:**
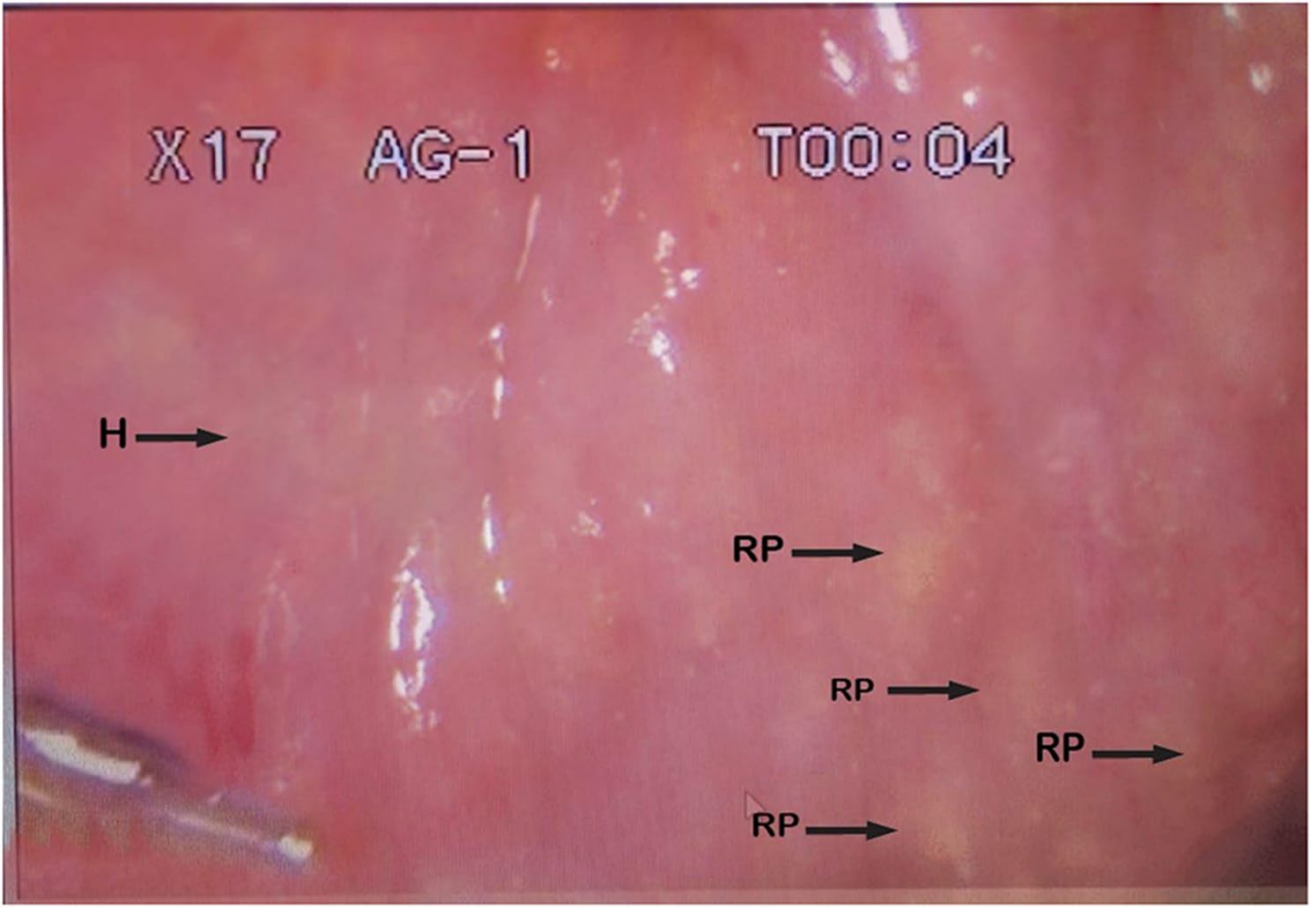
Colpophotograph of homogenous yellow sandy patches (H) and rubbery papules (RP) on the ectocervix

**Fig. 8:**
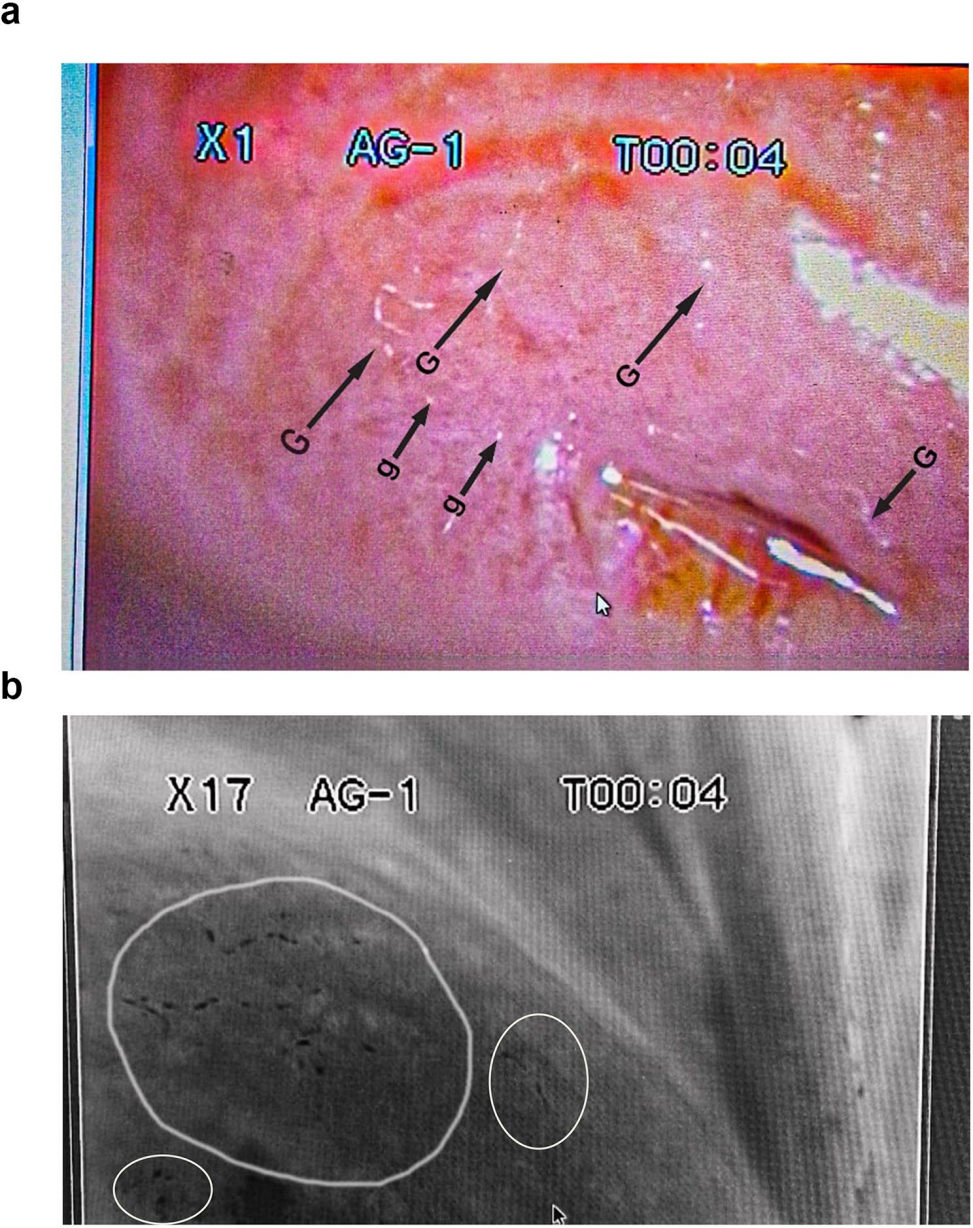
(**a**) Colpophotograph of grainy sandy patches (G), with single grains (g) on the ectocervix. (**b**) Monochrome colpophotograph showing areas on the ectocervix with grainy sandy patches

Of the 16.1% (40/248) females infected with *Schistosoma haematobium* (Table 2), heavy intensity (> 50 eggs/10 ml of urine) of urogenital schistosomiasis were recorded in 7.5% (3/40) (Table 3). Only the adolescent girls with a heavy intensity of urogenital schistosomiasis were further subjected to a colposcopy examination of the cervicovaginal canal to identify visible lesions of female genital schistosomiasis. Figures 6, 7 and 8a, and 8b shows the various lesions of female genital schistosomiasis identified.

**Fig. 9:**
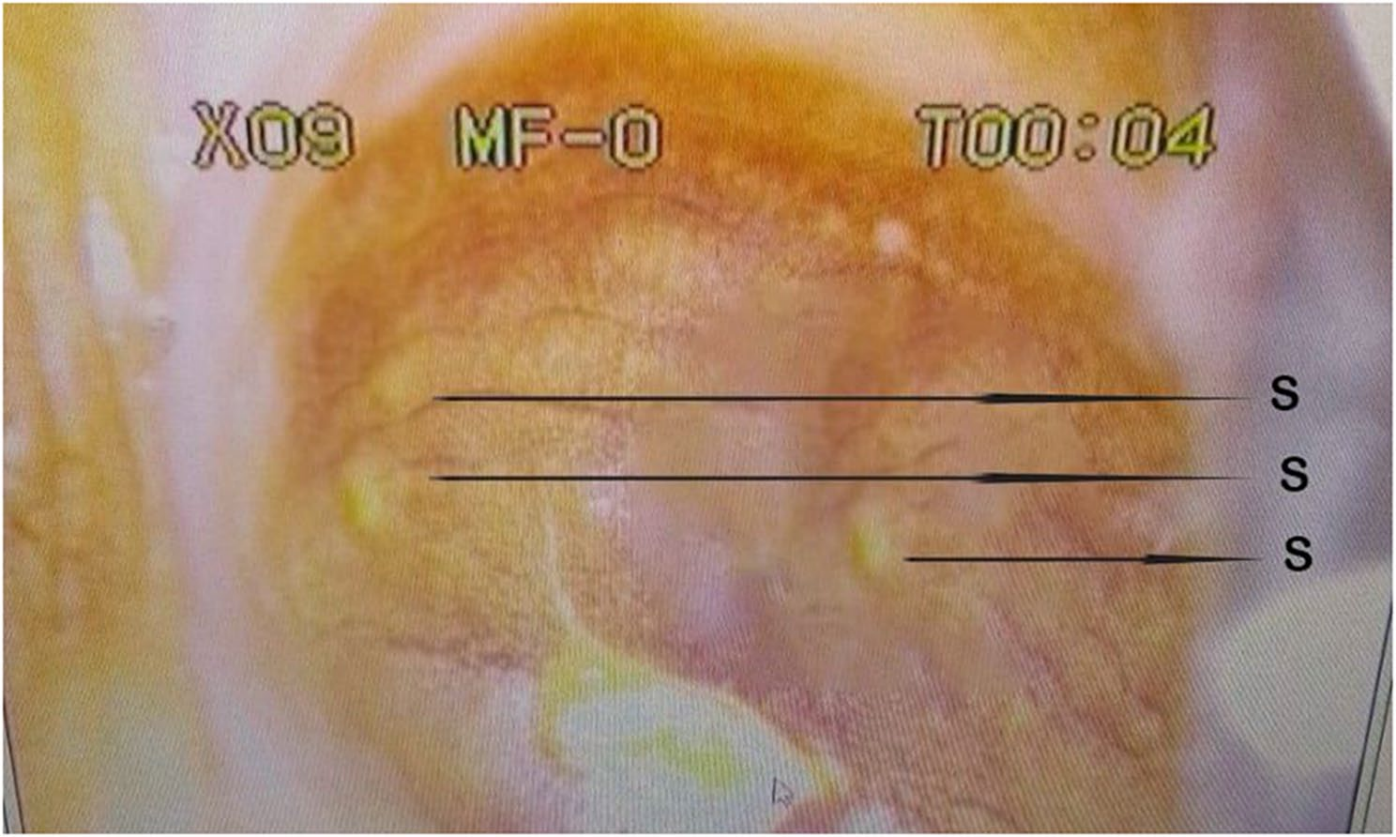
Colpophotograph of colpitis macularis or strawberry papules (S), found on the lateral fornix of the cervix indicating coinfection with *Trichomonas vaginalis*

Further analysis which involved the staining of the cervices with acetic acid showed no abnormal acetic whiteness and were therefore negative for precancerous lesions. However, a coinfection of female genital schistosomiasis and *Trichomonas vaginalis* was identified after staining the cervix with Lugol’s iodine solution (Fig. 9).

#### II. Presence of haematuria

**Fig. 10:**
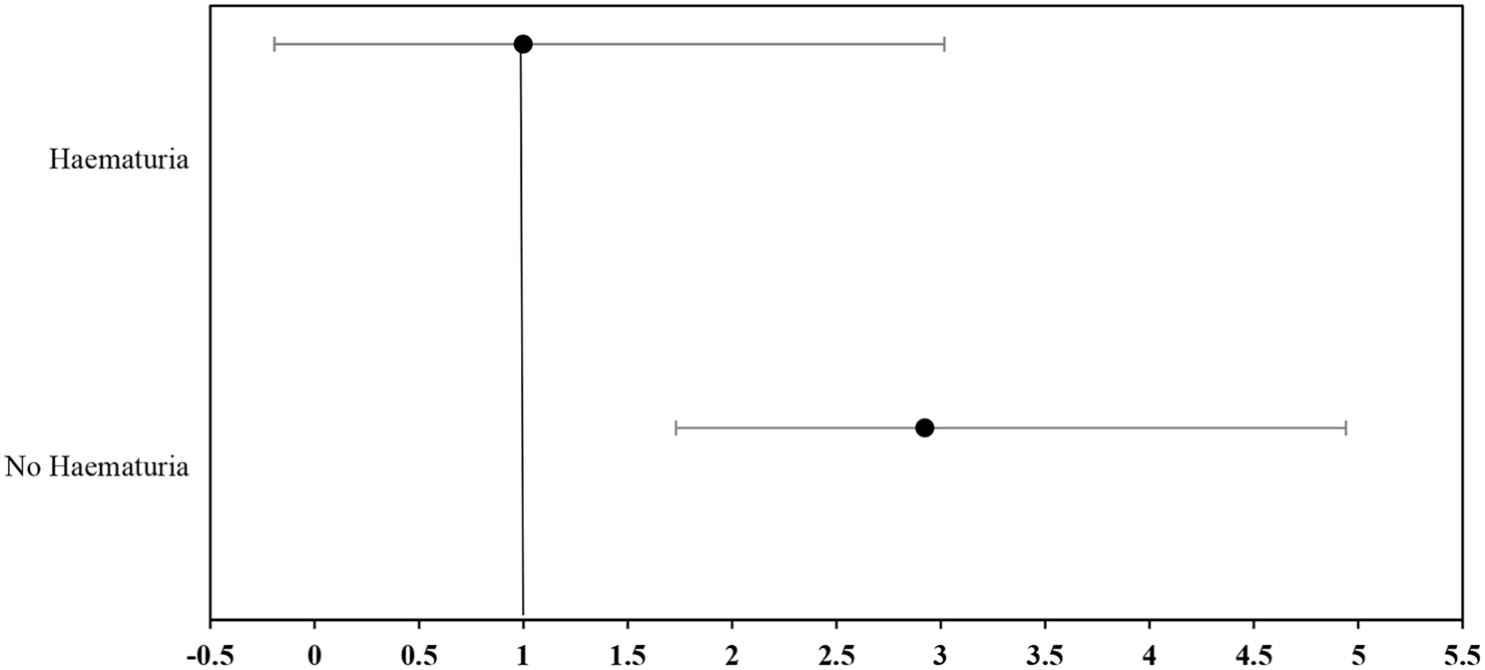
Forest plot showing the odds ratios for the association between presence of haematuria and urogenital schistosomiasis among adolescents studied

From the results, 30.2% (142/470) of the adolescents examined tested positive for haematuria (blood in the urine), while 69.8% (328/470) tested negative (Table 4). The highest prevalence of urogenital schistosomiasis was recorded among those who tested positive for haematuria at 24.6% (35/142), and this association was statistically significant (*p* < 0.05). The regression analysis showed that adolescents who tested negative for haematuria were 2.9 times more likely to be infected with urogenital schistosomiasis (95% CI: 1.731–4.941) (Fig. 10).

**Table 4:** Multinomial logistic analysis showing correlation between urogenital schistosomiasis and presence of haematuria

Presence of Haematuria	NE (%)	NN (%)	NI (%)	OR (95%CI)	p-Value
**No Haematuria**	328 (69.8)	295 (89.9)	33 (10.1)	2.924 (1.731–4.941)	0.000^*S*^
**Presence of Haematuria** ^**a**^	142 (30.2)	107 (75.4)	35 (24.60)	1.000	
**Total**	**470**	**402 (85.5)**	**68 (14.5)**		

NE = Number Examined. NN = Number Not infected. NI = Number Infected. OR = Odds Ratio. CI = Confidence Interval. a = Reference Category. S = Significant difference *p* < 0.05. (Multinomial Logistic Regression)

Again, 48.5% (33/68) of infected adolescents had no haematuria and recorded a mean intensity of 2.48eggs/10 ml of urine, no heavy infection and all had (100%) light intensity at 95% CI (1.8763–3.0934). Comparatively, those who tested positive for haematuria made up 51.5% (35/68) of the infected population. They recorded a light intensity in 91.4% (32/35) and the highest heavy intensity in 8.6% (3/35) at 95% CI (2.5825–13.1317), and the difference was statistically significant (*p* < 0.05) (Table 5).

**Table 5:** Mean intensity of urogenital schistosomiasis among the infected study population with respect to the presence of haematuria

Presence of Haematuria	NI (%)	Mean Interval ($\bar{x}$ ± σ) eggs/10 ml	Light Intensity (%)	Heavy Intensity (%)	95% CI	p-Value
No Haematuria	33 (48.5)	2.48(1.716)	33(100.0)	0	1.8763–3.0934	0.049^*S*^
Presence of Haematuria	35 (51.5)	7.85(15.354)	32(91.4)	3(8.6)	2.5825–13.1317	
Total	**68 (100)**	**5.25 (11.33002)**	**65 (95.6)**	**3 (4.4)**	**2.5076–7.9924**	

NI = Number Infected. CI = Confidence Interval. S = Significant difference *p* < 0.05 (Multinomial Logistic Regression)

#### III. Personal symptomatic reports

Table 6 shows the results of the personal symptomatic reports obtained from the in-depth questionnaire. Adolescents who experienced mild dysuria were 7.6 times more likely to be infected with urogenital schistosomiasis (OR: 7.619; 95% CI: 2.221–26.140; *p* < 0.05). The regression analysis showed a positive significant association between the experience of dysuria and urogenital schistosomiasis *(p* < 0.05).

**Table 6:** Multinomial logistic analysis showing correlation between urogenital schistosomiasis and personal symptomatic reports

Experience and severity of lower abdominal pain	NE	NI (%)	OR (95% CI)	p-value
No	152	35 (23.0)	0.453 (0.198–1.039)	0.061^*NS*^
Mild	120	14 (11.7)	1.027 (0.407–2.589)	0.956^*NS*^
Moderate	131	11 (8.4)	1.479 (0.565–3.873)	0.425^*NS*^
Severe^a^	67	8 (11.9)	1.000	
**Experience and severity of dysuria**				
No	319	41 (12.9)	6.027(2.201–16.502)	0.000^*S*^
Mild	67	7 (10.4)	7.619(2.221–26.140)	0.001^*S*^
Moderate	67	12 (17.9)	4.074(1.304–12.724)	0.016^*S*^
Severe^a^	17	8 (47.1)	1.000	
**Experience of haematuria**				
No	425	62 (14.6)	1.285 (0.376–4.396)	0.690^*NS*^
Yes^a^	45	6 (13.3)	1.000	
**Family history with haematuria**				
No	213	32 (15.0)	0.933(0.547–1.592)	0.798^*NS*^
Yes	38	5 (13.2)	1.088(0.395–3.001)	0.870^*NS*^
Not sure^a^	219	31 (14.2)	1.000	
**Experience and severity of itching or burning sensation in the genitals**				
No	207	35 (16.9)	2.184 (0.637–7.492)	0.214^*NS*^
Mild	106	16 (15.1)	2.500 (0.687–9.103)	0.165^*NS*^
Moderate	144	13 (9.0)	4.479(1.210–16. 573)	0.025^*S*^
Severe^a^	13	4 (30.8)	1.000	
**Experience of abnormal genital discharge**				
No	269	43 (16.0)	1.415 (0.578–3.467)	0.448^*NS*^
Yes	168	18 (10.7)	2.244 (0.853–5.902)	0.102^*NS*^
Sometimes^a^	33	7 (21.2)	1.000	
**Experience of dyspareunia**				
Not applicable	418	61 (14.6)	1.672 (0.339–8.239)	0.527^*NS*^
Rarely	31	5 (16.1)	1.486 (0.236–9.355)	0.673^*NS*^
Occasionally	12	0 (0.0)		
Always^a^	9	2 (22.2)	1.000	
**Total**	**470**	**68 (14.5)**		

NE = Number Examined. NI = Number Infected. OR = Odds Ratio. CI = Confidence Interval. a = Reference Category. S = Significant difference *p* < 0.05. NS = No Significant difference *p* > 0.05 (Multinomial Logistic Regression)

The results also showed that adolescents who reported that they had not experienced haematuria recorded the highest prevalence of 14.6% (62/425). This finding was not statistically significant; however, they had higher odds of being infected with *Schistosoma haematobium* (OR: 1.285; 95% CI: 0.376–4.396; *p* > 0.05). Additionally, the highest prevalence of 15.0% (32/213) was recorded among adolescents who reported no family history of haematuria. This finding was not statistically significant (*p* > 0.05).

With respect to the experience of itching or burning sensation in the genitals, adolescents who experienced severe itching or burning sensation in the genitals had the highest prevalence of 30.8% (4/13). Adolescents who experienced moderate itching or burning sensation in the genitals recorded the lowest prevalence of 9.0% (13/144). This disparity was found to be statistically significant (*p* < 0.05), and the regression analysis showed that they were 4.5 times more likely to be infected with urogenital schistosomiasis (OR: 4.479; 95% CI: 1.210-16.573). Notably, those who always experienced dyspareunia had the highest prevalence of 22.2% (2/9).

Among the females infected with UgS (40/68), 7.5% (3/40) were examined and found positive for FGS. 33.3% (1/3) of the FGS-infected females experienced lower abdominal pain. The chi-square analysis showed that dysuria and itching/burning sensation in the genitals were statistically significant with FGS (*p <* 0.05) (Table 7).

**Table 7:** Prevalence of Female Genital Schistosomiasis with respect to Personal Symptomatic Reports (*N* = 40)

Experience and severity of lower abdominal pain	NE	NI (%)	X^2^	p-value
No	16 (40.0)	0 (0.0)	7.327	0.062^*NS*^
Mild	11 (27.5)	0 (0.0)		
Moderate	10 (25.0)	2 (20.0)		
Severe	3 (7.5)	1 (33.3)		
**Experience and severity of dysuria**				
No	22 (55.0)	0 (0.0)	8.769	0.033^*S*^
Mild	3 (7.5)	1 (33.3)		
Moderate	7 (17.5)	0 (0.0)		
Severe	8 (20.0)	2 (25.0)		
**Experience of haematuria**				
No	34 (85.0)	3 (8.8)	0.572	0.449^*NS*^
Yes	6 (15.0)	0(0.0)		
**Family history with haematuria**				
No	14 (35.0)	0 (0.0)	2.934	0.231^*NS*^
Yes	5 (12.5)	0 (0.0)		
Not sure	21 (52.5)	3 (14.3)		
**Experience and severity of itching or burning sensation in the genitals**				
No	26 (65.0)	0 (0.0)	7.568	0.023^*NS*^
Mild	12 (30.0)	3 (25.0)		
Moderate	2 (5.0)	0 (0.0)		
Severe	0 (0.0)	0 (0.0)		
**Experience of abnormal genital discharge**				
No	27 (67.5)	3 (11.1)	1.562	0.458^*NS*^
Yes	11 (27.5)	0 (0.0)		
Sometimes	2 (5.0)	0 (0.0)		
**Experience of dyspareunia**				
Not applicable	38 (95.0)	3 (7.9)	0.171	0.679^*NS*^
Rarely	2 (5.0)	0 (0.0)		
**Total**	**40 (100)**	**3 (7.5)**		

NE = Number Examined. NI = Number Infected. X^*2*^ = Chi-square value. S = Significant difference *p* < 0.05. NS = No Significant difference *p* > 0.05 (Chi-square test)

#### Socio-economic factors

Among the adolescents examined for their religious affiliation, a striking 99.6% identified as Christians, while only 0.42% practiced African traditional religion. Notably, the presence of urogenital schistosomiasis was exclusively observed among Christian participants, with a prevalence of 14.5%. However, this disparity was not statistically significant (*p* > 0.05) (Table 8). Regarding the occupations of their parents, the highest prevalence of 28.6% was recorded among adolescents whose parents worked in public or civil service roles. Conversely, adolescents with parents or guardians engaged in trade exhibited a statistically significant prevalence of 13.1% (*p* < 0.05). Regression analysis indicated that they were approximately 2.5 times more likely to be infected with urogenital schistosomiasis compared to those with professional parents (OR: 2.484; 95% CI: 1.078–5.727). Regarding the literacy level of parents, the study revealed no significant association with urogenital schistosomiasis (*p* > 0.05).

**Table 8:** Multinomial logistic analysis showing correlation between urogenital schistosomiasis and socio-economic factors (*N* = 470)

	Category	NE	NI (%)	OR (95% CI)	p-value
**Religion**	**ATR**	2 (0.42)	0 (0.0)	X	0.999^*NS*^
	**Christianity** ^**a**^	468 (99.6)	68 (14.5)	1.000	
**Parents’ occupation**	**Traders/Entrepreneurs**	305 (64.9)	40 (13.1)	2.484 (1.078–5.727)	0.033^*S*^
	**Public/ Civil Servant**	21 (4.5)	6 (28.6)	0.937 (0.277–3.169)	0.917^*NS*^
	**Teachers**	13 (2.8)	0 (0.0)		
	**Farmers**	69 (14.7)	9 (13.0)	2.500 (0.885–7.060)	0.084^*NS*^
	**Drivers**	29 (6.1)	4 (13.80)	2.344 (0.636–8.636)	0.201^*NS*^
	**Professionals**	33 (7.0)	9 (27.3)	1.000	
**Parents’ Literacy Level**	**No formal Education**	15 (3.2)	3 (20.0)	0.620 (0.150–2.553)	0.508^*NS*^
	**Primary Education**	80 (17.2)	8 (10.0)	1.394 (0.530–3.671)	0.50I^*NS*^
	**Secondary Education**	293 (62.3)	46 (15.7)	0.832 (0.409–1.690)	0.611^*NS*^
	**Tertiary Education** ^**a**^	82 (17.3)	11 (13.4)	1.000	
**Water facility in school**	**Not available**	284 (60.4)	38 (13.4)	1.245 (0.741–2.092)	0.408^*NS*^
	**Available** ^**a**^	186 (39.6)	30 (16.1)	1.000	
**Water facility in the home**	**Not available**	24 (5.1)	5 (20.8)	0.856 (0.278–2.637)	0.787^*NS*^
	**Poorly available**	35 (7.4)	6 (17.1)	1.089 (0.388–3.060)	0.871^*NS*^
	**Fairly available**	114 (24.3)	5 (4.4)	4.913(1.723 − 14.007)	0.003^*S*^
	**Available**	210 (44.7)	36 (17.1)	1.089 (0.568–2.087)	0.797^*NS*^
	**Always available** ^**a**^	87 (18.5)	16 (18.4)	1.000	
**Source of Drinking Water**	**Borehole (Tap)**	237 (50.4)	9 (3.8)	9.806 (3.822–25.159)	0.00^*S*^
	**Freshwater body**	21 (4.5)	4 (19.0)	1.645 (0.459–5.899)	0.445^*NS*^
	**Sachet or Bottled Water**	50 (10.6)	7 (14.0)	2.378 (0.840–6.729)	0.103^*NS*^
	**Borehole and Freshwater body**	79 (16.8)	29 (36.7)	0.667 (0.297–1.498)	0.327^*NS*^
	**Borehole and Sachet/Bottled Water**	40 (8.5)	7 (17.5)	1.825 (0.637–5.232)	0.263^*NS*^
	**All Water Sources** ^**a**^	43 (9.2)	12 (27.9)	1.000	

NE = Number Examined. NI = Number Infected. OR = Odds Ratio. CI = Confidence Interval. a = Reference Category. X = Undefined values due to 0.0% prevalence of infection S = Significant difference *p* < 0.05. NS = No Significant difference *p* > 0.05 (Multinomial Logistic Regression)

An evaluation of water facilities, both at home and the school, unveiled noteworthy insights regarding access, quality, and infrastructure. Adolescents with moderately available home water facilities displayed a significant prevalence of 4.4% (p < 0.05). The term ‘moderately available’ denotes instances where access to water may experience occasional interruptions or inconsistencies, though not to the extent of being consistently unavailable. Regression analysis further indicated that the adolescents in these households were 4.9 times more susceptible to urogenital schistosomiasis compared to those with consistently available home water (OR: 4.931; 95% CI: 1.723–14.007). In comparison, adolescents with school water facilities exhibited a higher prevalence of 16.10%, while those lacking such facilities recorded 13.4%. No significant disparity emerged concerning the source of water (*p* > 0.05). With regards to the source of drinking water, the participants identified 3 primary water sources, where some had dual or more sources of water. The highest prevalence of 36.7% (29/79) was recorded among those who drank from boreholes and freshwater bodies like streams, rivers, lakes, ponds, and spring. However, those who got their water from boreholes had the lowest prevalence at 3.8% (9/237) but were more likely to be infected (OR: 9.806; 95% CI: 3.822–25.159), and this finding was statistically significant (*p* < 0.05). Additionally, participants who drank packed water (sachet or bottled water), had an increased likelihood of infection (OR: 2.378; 95% CI: 0.840–6.729; p value: 0.103). The ethnographic interviews highlighted the challenges faced by some participants in accessing clean water.

We do have water at home, but it’s not from a borehole… So, after selling vegetables by the riverside, we just fetch water from the river. It’s more convenient that way.” (Female participant A, SS 1, 15–19 years old).

Digisa is spring water. It flows from the rock, and the water tastes really good. When we can’t afford water from the borehole, we simply fetch from there.” (Male participant A, 10–14 years old).

For the past two years, there’s been no water in our school. We often have to buy water from the neighboring compound where they have running water.” (Male participant B, 10–14 years old).

### Water contact and water contact activities

Out of the 470 adolescents examined, 52.3% (246/470) were certain that they had direct contact with freshwater bodies, while 5.4% (25/470) were unsure, and 42.3% (199/470) were certain that they had not had such contact. This direct contact with water bodies was found to have a significant positive association with the risk of infection (*p* < 0.05). Regression analysis revealed that those with direct contact were 2.6 times more likely to be infected with urogenital schistosomiasis (Table 9).

**Table 9:** Multinomial logistic analysis showing correlation between urogenital schistosomiasis and behavioural factors (water contact and water contact activities) (*N* = 470)

	Category	NE	NI (%)	OR (95% CI)	p-value
**Visit to freshwater body**	Yes	271	39 (14.4)	1.015 (0.604–1.706)	0.956^*NS*^
	No^a^	199	29 (14.6)	1.000	
**Direct contact with freshwater body**	Yes	246	32 (13.0)	2.601 (1.007–6.716)	0.048^*S*^
	No	199	29 (14.6)	2.280 (0.875–5.940)	0.092^*NS*^
	Not sure^a^	25	7 (28.0)	1.000	
**Purpose of visiting waterbody**	No answer	129	14 (10.9)	6.389 (2.058–19.835)	0.001^*S*^
	Domestic purposes	78	5 (6.4)	11.356 (2.972–43.382)	0.000^*S*^
	Recreational purposes	247	42 (17.0)	3.796 (1.339–10.762)	0.012^*S*^
	Occupational purposes^a^	16	7 (43.7)	1.000	
	No answer	129	14 (10.9)	6.389 (2.058–19.835)	0.001^*S*^
**Duration of time spent in waterbody**	No answer	139	17 (12.2)	0.567 (0.243–1.322)	0.189^*NS*^
	30 min	130	20 (15.4)	0.434 (0.189–0.995)	0.049^*S*^
	1 h	78	22 (28.2)	0.201 (0.087–0.465)	0.000^*S*^
	Above 1 hour^a^	123	9 (7.3)	1.000	

NE = Number Examined. Not infected. NI = Number Infected. OR = Odds Ratio. CI = Confidence Interval. a = Reference Category. S = Significant difference *p* < 0.05. NS = No Significant difference *p* > 0.05 (Multinomial Logistic Regression)

**Fig. 11:**
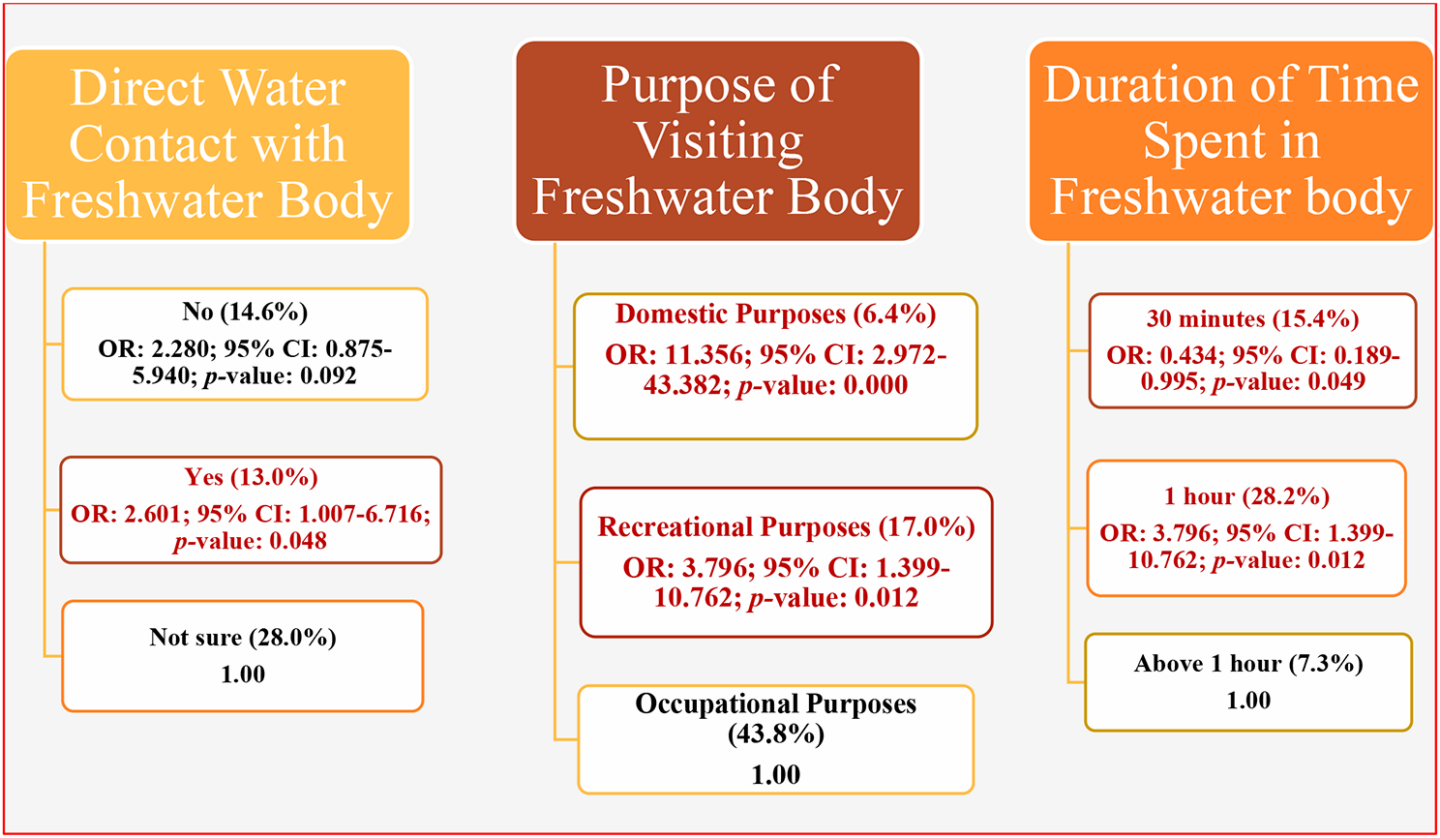
Behavioural factors (water contact and water contact activities) associated with urogenital schistosomiasis

The reasons given by adolescents for visiting freshwater bodies were found to be statistically significant (*p* < 0.05). Notably, the highest prevalence of urogenital schistosomiasis (43.8%) was observed among those visiting for occupational purposes. However, adolescents who visited freshwater bodies for domestic purposes showed even higher odds of infection (OR: 11.356; 95% CI: 2.972–43.382) (Fig. 11).

**Fig. 12:**
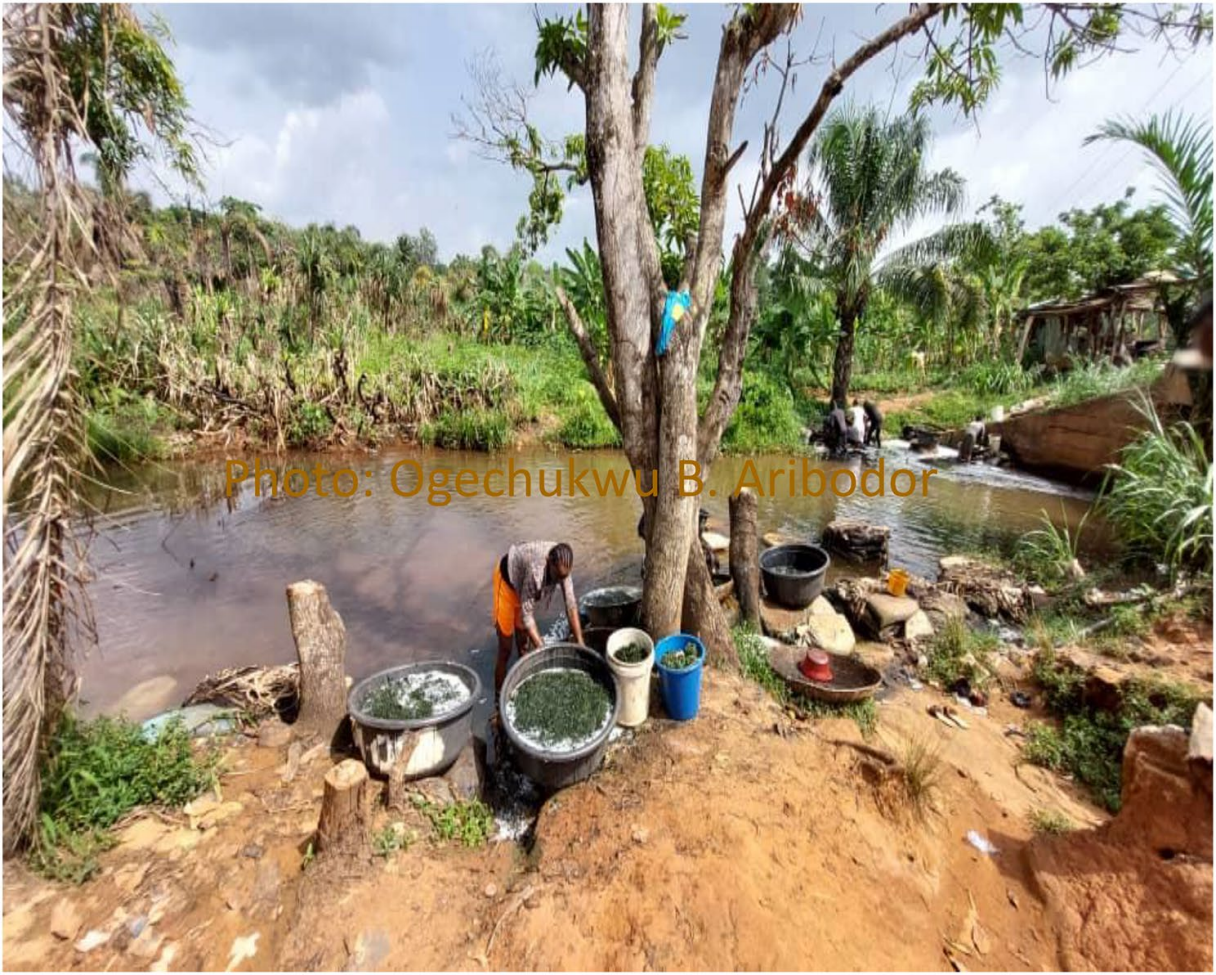
Adolescents in direct contact with freshwater body in the study area for domestic and occupational purposes

Furthermore, ethnographic interviews and observations (Figs. 11 and 12) provided additional context to these findings. For example, one teenager mentioned,

Yes. Me I like going to the river close to the school when school is over. I stop to buy snacks from the shops at the riverbanks. I have entered the water, but just to wash my hands, not to swim (Female B participant, 10–14 years old).

This interview aligns with the observation that adolescents who visited water bodies for many several reasons, including recreational activities, had an increased risk of urogenital schistosomiasis.

Similarly, another teenager explained,

My mother sells bitter leaf at the riverbank. I go with her to help her wash the vegetables before selling (Female C participant, 15–19 years old).

This statement corresponds to the finding that occupational visits to freshwater bodies were associated with a high prevalence of urogenital schistosomiasis (Fig. 12).

Moreover, interviews with adolescents who worked as Keke Napep (tricycle) drivers shed light on their interaction with freshwater bodies.

I am also a Keke driver. I use it to put myself through school. It is easier to wash my keke at the rivers. Everybody goes there too. You meet people and gist while doing whatever you want. If the weather is too hot, I take a swim (Male participant C, 15–19 years old), one teenager explained.

The duration of stay in freshwater bodies showed a positive association with the risk of urogenital schistosomiasis (*p* < 0.05), with the highest prevalence (28.2%) observed among individuals who stayed in the water for 1 h. Additionally, apart from the risk of UgS, some adolescents with UgS presented with maculopapular skin lesions consistent with cercarial dermatitis or swimmers’ itch. Syndromic evaluations indicated that these individuals also experienced mild fever and severe itching, leading to excoriations consistent with cercarial dermatitis. The observed lesions resembled those in the healing stages, with patches of pigmented skin. The observed lesions were on the legs and arms. Furthermore, Knowledge, Attitude, and Practice (KAP) evaluations indicated that the affected individuals not only had a history of water contact but also reported recent direct contact with freshwater bodies, up to two weeks preceding the observation. However, further confirmatory tests to identify any other potential underlying causes of the rashes were not conducted. One of the reports states,

My mother is from Inoma (an endemic riverine community 58km away from the study area). Before the flood came in last year, we used to visit every Christmas and swim in the river. But sometimes when I go into the water, I get these boils and they itch and wound me. They say its Ahu mmili, that my body does not like water. It happens to all my younger siblings too. This one was from last week Saturday when I went to fetch water from the river.” (Female participant D, 15–19 years old).

### Knowledge of diseases and attitude to treatment

The findings presented in Table 10 indicated a notable portion of participants lacked awareness of urogenital schistosomiasis (44.9%) and female genital schistosomiasis (95.3%). Interestingly, individuals with limited awareness exhibited the highest likelihood of urogenital schistosomiasis, with statistical significance (OR: 3.701; 95% CI: 1.509–9.076; *p* < 0.05).

**Table 10:** Multinomial logistic analysis showing correlation between urogenital schistosomiasis and behavioural factors (knowledge of infection and attitude to treatment) (*N* = 470)

	Category	NE	NI (%)	OR (95% CI)	p-value
**Knowledge of urogenital schistosomiasis and source of knowledge**	No	211 (44.9)	21 (10.0)	3.701(1.509–9.076)	0.004^*S*^
	School	162 (34.5)	26 (16.0)	2.140(0.886–5.168)	0.091^*NS*^
	Church	38 (8.0)	5 (13.20)	2.700(0.798–9.137)	0.110^*NS*^
	Mass media	28 (6.0)	7 (25.0)	1.227(0.387–3.894)	0.728^*NS*^
	Mass Administration Medicines^a^	31 (6.6)	9 (29.0)	1.000	
**Knowledge of female genital schistosomiasis**	No	448 (95.3)	62 (13.8)	2.335 (0.880–6.195)	0.089^*NS*^
	Yes^a^	22 (4.7)	6 (27.3)	1.000	
**Knowledge of transmission of urogenital schistosomiasis**	No idea	454 (96.6)	65 (14.3)	2.565(0.647–10.172)	0.180^*NS*^
	Vector borne route	4 (0.9)	0 (0.0)	X	X
	Oral-faecal route	2 (0.4)	0 (0.0)	X	X
	Skin penetration^a^	10 (2.1)	3 (30.0)	1.000	
**Knowledge of transmission of female genital schistosomiasis**	No idea	461 (98.1)	65 (14.1)	9.138 (1.498–55.746)	0.016^*S*^
	Vector borne route	4 (0.9)	0 (0.0)	X	X
	Oral-faecal route^a^	5 (1.0)	3 (60.0)	1.000	
**Treatment with Praziquantel and where it was administered**	No answer	161 (34.2)	29 (18.0)	0.845(0.413–1.729)	0.645^*NS*^
	Over the counter	196 (41.7)	19 (9.7)	1.730(0.811–3.691)	0.156^*NS*^
	Hospital	30 (6.4)	7 (23.3)	0.610(0.217–1.714)	0.348^*NS*^
	Mass Administration of Medicines^a^	83 (17.7)	13 (15.70)	1.000	
**Duration of last deworming**	No answer	10 (2.1)	0 (0.0)	X	X
	1–3 months	39 (8.3)	5 (12.8)	1.040 (0.388–2.784)	0.938^*NS*^
	4–6 months	44 (9.4)	13 (29.5)	0.365 (0.179–0.744)	0.006^*S*^
	Above 6 months^a^	377 (80.2)	50 (13.3)	1.000	
**Individual who administered the medicine**	No answer	72 (15.3)	16 (22.2)	0.496 (0.236–1.043)	0.064^*NS*^
	Family member	253 (53.8)	34 (13.4)	0.913 (0.495–1.683)	0.770^*NS*^
	Health practitioner^a^	145 (30.9)	18 (12.4)	1.000	
**Compliance with height-based dosage**	No	257 (54.7)	42 (16.3)	0.602 (0.203–1.787)	0.361^*NS*^
	Yes	175 (37.2)	22(12.6)	0.818 (0.265–2.529)	0.727^*NS*^
	Not sure^a^	38 (8.1)	4 (10.5)	1.000	
**Comfort level discussing genital health challenges**	Not comfortable	116 (24.7)	21 (18.1)	0.251 (0.083–0.764)	0.015^*S*^
	Very comfortable	278 (59.1)	43 (15.5)	0.304 (0.105–0.875)	0.027^*S*^
	Somewhat comfortable^a^	76 (16.2)	4 (5.3)	1.000	
**Preferred individual for discussing such issues**	Family member	305 (64.9)	40 (13.1)	1.237 (0.639–2.394)	0.528^*NS*^
	Sibling	46 (9.8)	3 (6.5)	2.676 (0.728–9.838)	0.139^*NS*^
	Friend	23 (4.9)	9 (39.1)	0.290 (0.105–0.800)	0.017^*S*^
	Teacher	7(1.5)	2 (28.6)	0.467 (0.082–2.649)	0.390^*NS*^
	Health practitioner^a^	89 (18.9)	14 (15.7)	1.000	
**Importance of prevention and treatment of urogenital schistosomiasis**	Not important	9 (1.9)	0 (0.0)	X	X
	Very important	356 (75.7)	56 (15.7)	0.691 (0.355–1.345)	0.277^*NS*^
	Somewhat important^a^	105 (22.4)	12 (11.4)	1.000	
**Importance of prevention and treatment of female genital schistosomiasis**	Not important	9 (1.9)	0 (0.0)	X	X
	Very important	356 (75.7)	58 (16.3)	0.541 (0.266–1.100)	0.090^*NS*^
	Somewhat important^a^	105 (22.4)	10 (9.5)	1.000	

NE = Number Examined. NI = Number Infected. OR = Odds Ratio. CI = Confidence Interval. a = Reference Category. X = undefined values due to 0.0% prevalence of infection

S = Significant difference *p* < 0.05. NS = No Significant difference *p* > 0.05 (Multinomial Logistic Regression)

Regarding knowledge about the transmission of these diseases, the study indicated that adolescents who were aware that urogenital schistosomiasis is transmitted through skin penetration by cercariae had the highest prevalence of urogenital schistosomiasis at 30.0% (3/10). Conversely, those who had no knowledge of how these diseases are transmitted also had a notable prevalence of urogenital schistosomiasis.

When it comes to treatment, the study did not find a significant association between praziquantel treatment and urogenital schistosomiasis. Similarly, the location where treatment was administered did not show a significant link to prevalence of urogenital schistosomiasis. However, there was a significant association with the duration of last deworming and urogenital schistosomiasis, as adolescents who had been dewormed in the last 4–6 months had a higher prevalence of infection at 29.5% (13/44).

The individuals who administered the medicine did not appear to have a significant impact on the prevalence of urogenital schistosomiasis. However, those who received treatment from a family member had a slightly higher prevalence at 13.4% (34/253).

A female participant D, aged 10–14 years old reported,

I have heard of okpo mamili (Urogenital Schistosomiasis). Those nurses that share drugs in primary schools, gave them to us. That was in 2019 or when I was in primary 5. But I did not know that it can affect the private part.

In contrast, another Male participant D, 10–14 years old, expressed a lack of awareness, saying,

I have not heard of it, but my mother gives us ogwu okpo (anthelminthics). She does not measure our height or weight when she gives it to us.

In terms of comfort levels with discussing genital-related health challenges, the study found a significant association with the prevalence of urogenital schistosomiasis. Adolescents who were more comfortable discussing such issues with friends had the highest prevalence of urogenital schistosomiasis at 39.1% (9/23). This aligns with an ethnography interview where a female participant (E, 15–19 years old) explained,

I can’t talk to my mother about my private part; she will not understand. I am very close to my school mother. She is in SS 3 now. She is older and more experienced. It is easier than talking to my mother.

Another participant (Male participant E, 15–19 years old) shared,

I do not live with my parents, so I cannot talk to them. My sponsors barely treat me when I have malaria. If I tell them my private part itches me, they will just say I am dirty.

Yet another participant reported,

Nurses in the health center know my parents. They are also from this community. If I tell them anything, they will tell my parents and other elders. I don’t want my business to become public property.” (Male participant F, 15–19 years old).

Nurses in the health center know my parents. They are also from this community. If I tell them anything, they will tell my parents and other elders. I don’t want my business to become public property.” (Male participant F, 15–19 years old).

Notably, a substantial majority (75.7%) of the study participants (356/470) believed that preventing and treating urogenital schistosomiasis and female genital schistosomiasis were important. An interviewee (Female participant F, 15–19 years old) stated,

Yes, I think it is very important that people know about okpo mamili, especially since it can affect the private part. I did not even know that it is not gotten through sex. Giving us medicines is not enough. I cannot even remember the last time I took the drugs. They should let us know what to do and how we can help ourselves.

(Table 11)

Table 11 showed that the highest prevalence of FGS among females with urogenital schistosomiasis, who did not know about either UgS or FGS at a prevalence of 17.6% (3/17) and 8.1% (3/37) respectively. Additionally, infected females were last dewormed 4–6 moths before the study was carried out (10.3%; 3/29). The chi-square analysis showed no significant relationship between behavioural factors (knowledge of infection and attitude to treatment) and FGS (*p >* 0.05).

**Table 11:** Prevalence of female genital schistosomiasis with respect to behavioural factors (knowledge of infection and attitude to treatment) (*N* = 40)

	Category	NE	NI (%)	X^2^	p-value
**Knowledge of urogenital schistosomiasis and source of knowledge**	No	17 (42.5)	3 (17.6)	4.388	0.356^N*S*^
	School	15 (37.5)	0 (0.0)		
	Church	2 (5.0)	0 (0.0)		
	Mass media	2 (5.0)	0 (0.0)		
	Mass Administration Medicines^a^	4 (10)	9 (0.0)		
**Knowledge of female genital schistosomiasis**	No	37(92.5)	3 (8.1)	0.263	0.608^*NS*^
	Yes^a^	3 (7.5)	0 (0.0)		
**Knowledge of transmission of urogenital schistosomiasis**	No idea	40(100)	3 (7.5)		
	Vector borne route	0 (0.0)	0 (0.0)		
	Oral-faecal route	0 (0.0)	0 (0.0)		
	Skin penetration^a^	0 (0.0)	0 (0.0)		
**Knowledge of transmission of female genital schistosomiasis**	No idea	37 (92.5)	3 (8.1)	0.263	0.608^*NS*^
	Vector borne route	0 (0.9)	0 (0.0)		
	Oral-faecal route^a^	3 (7.5)	0 (0.0)		
**Treatment with Praziquantel and where it was administered**	No answer	18 (45.0)	3 (16.7)	3.964	0.265^NS^
	Over the counter	12 (30.0)	0 (0.0)		
	Hospital	4 (10)	0 (0.0)		
	Mass Administration of Medicines	6 (15.0)	0 (0.00)		
**Duration of last deworming**	No answer	5 (12.5)	0 (0.0)	1.230	0.541^NS^
	1–3 months	6 (15.0)	0 (0.0)		
	4–6 months	29 (72.5)	3 (10.3)		
**Individual who administered the medicine**	No answer	14 (35.0)	0 (0.0)	3.243	0.198^*NS*^
	Family member	20 (50.0)	3 (15.0)		
	Health practitioner	6 (15.0)	0 (0.0)		
**Compliance with height-based dosage**	No	22 (55.0)	3 (13.6)	2.654	0.265^*NS*^
	Yes	16 (40.0)	0 (0.0)		
	Not sure	2 (5.0)	0 (0.0)		
**Comfort level discussing genital health challenges**	Not comfortable	10 (25.0)	0 (0.0)	1.746	0.418^*NS*^
	Very comfortable	26 (65.0)	3 (11.5)		
	Somewhat comfortable	4 (10.0)	0 (0.0)		
**Preferred individual for discussing such issues**	Parents	24 (60.0)	3 (12.5)	2.162	0.706^*NS*^
	Sibling	3 (7.5)	0 (0.0)		
	Friend	3 (7.5)	0 (0.0)		
	Teacher	2 (5.0)	0 (0.0)		
	Health practitioner	8 (20.0)	0 (0.0)		
**Importance of prevention and treatment of urogenital schistosomiasis**	Not important	0 (0.0)	0 (0.0)	0.360	0.548^*NS*^
	Very important	36 (90.0)	3 (8.3)		
	Somewhat important	4 (10.0)	0 (0.0)		
**Importance of prevention and treatment of female genital schistosomiasis**	Not important	0 (0.0)	0 (0.0)	0.360	0.548^*NS*^
	Very important	36 (90.0)	3 (8.3)		
	Somewhat important	4 (10.0)	0 (0.0)		

NE = Number Examined. NI = Number Infected. X^2^ = Chi-square value. S = Significant difference *p* < 0.05. NS = No Significant difference *p* > 0.05 (Chi-square test)

## Discussion

The overall prevalence of urogenital schistosomiasis among adolescents studied in Anambra State was 14.5%, with no statistically significant difference between genders. However, adolescents aged 10–14 years exhibited the highest prevalence, which may be attributed to their increased involvement in water-related activities as adduced by previous studies [24, 65, 66]. This rate is notably higher compared to recent studies within Anambra State and indicates the need for revised intervention strategies [10, 27, 49]. The exclusion of secondary school adolescents from current mass administration of medicines (MAMs) programmes, which mainly target primary school-aged children, may have contributed to this prevalence. Previous studies had also shown that this selective approach hampers control efforts, as infected adults and adolescents serve as reservoirs for transmission to vulnerable children [47, 67, 68]. Additionally, the overall prevalence of UgS in comparison to other regions of Nigeria where MAMs successfully covered school-aged children (5–14 years) was lower in these studies [21, 23, 24, 69, 70]. This highlights the effectiveness of MAMs that has been ongoing in Anambra State for a decade but also underlines the necessity for a more comprehensive, long-term control strategy to achieve complete eradication.

Most cases of urogenital schistosomiasis seen in this study were of light intensity, with heavy intensity of UgS observed in a smaller proportion of cases, particularly among females and older adolescents. Occupational visits and domestic purposes involving freshwater bodies posed higher risks. Additionally, longer durations of stay in freshwater bodies increased the risk of urogenital schistosomiasis especially among the female adolescents. Limited or irregular access to available home water facilities was also associated with a higher prevalence of UgS, highlighting the importance of consistent access to clean water sources. This finding aligns with previous studies in the area, indicating the importance of gender-sensitive interventions [3, 10]. Among females infected with *S. haematobium*, those with heavy intensity were further examined for visible lesions of female genital schistosomiasis. Coinfection with *Trichomonas vaginalis* was identified in some cases. This study confirmed female genital schistosomiasis (FGS), which is consistent with the findings of Aribodor et al. [10] in Nsugbe community of Anambra State and co-infection of FGS with *Trichomonas vaginalis* in Anaocha LGA. This agrees with a previous study by Sturt et al. [36] that also recorded that a higher percentage of FGS-positive women in Zambia were also infected with *Trichomonas vaginalis.* Despite increased efforts, clinical surveillance for FGS in Nigeria remains notably deficient. This monitoring gap can be attributed to factors such as a lack of awareness and limited experience among healthcare personnel at various levels [71]. The scarcity of resources for FGS detection in primary care settings, exemplified in the study area where only one diagnostic equipment was available, further compounds the challenge.

Moreover, conflicting priorities within healthcare programs, where more visible and well-known diseases often take precedence, exacerbate the issue. Previous study conducted in Anambra state highlights this disparity, revealing that while more health professionals were well-informed about schistosomiasis, awareness of FGS was notably lacking [18]. Furthermore, previous studies have shown that FGS infections are often associated and potentially increase the risk of sexually transmitted genital diseases [36, 40] due to the development of lesions and ulcers in the female genital organs, heightening vulnerability to the transmission of infections during sexual activity. The damage inflicted by schistosome eggs in the genital tissues compromises the natural barrier function, facilitating the entry of various pathogens, including those causing sexually transmitted infections (STIs). The immune response triggered by the presence of schistosome eggs creates an environment conducive to the replication and transmission of additional infectious agents, increasing the risk of acquiring further infections.

There were no significant associations found between religion, parents’ literacy, and UgS status, however, adolescents whose parents were traders/entrepreneurs were significantly associated with UgS. This highlights that water contact is influenced by community norms and behaviours [15]. Drinking from boreholes was significantly associated with UgS, while those who drank from sachet/bottled water were 2 times more likely to be infected with UgS. This discovery is unique as there is no evidence on the transmission of urogenital schistosomiasis through drinking water. However, this emphasizes the need to ensure the safety of commercial water sources. This finding showed that there is a behavioural challenge as even when safe water is provided, some adolescents might use both a borehole for home and visit freshwater bodies for work or fun. Coinfection of UgS with cercarial dermatitis was observed in adolescents with a history of water contact. The skin rash is also common in the cultural context, where the infection is known as ‘ahu mmili’ (meaning ‘body of water’ in the local Igbo dialect. This points to a deeper environmental problem, as water bodies may be heavily infested with schistosomes to an extent that hybrid species may also be present, indicating the need for alternative safe water sources and vector control measures. Furthermore, the study observed the clinical manifestation of cercarial dermatitis in some infected adolescents, which is often overlooked by the community and healthcare workers. Implementing a behavioral change communication system involving community members, including traditional healers, may help to address this issue.

The study has some limitations that need to be considered for a comprehensive understanding of its findings. Firstly, the study’s geographical scope was limited to Anaocha Local Government Area (LGA) of Anambra State, which may not fully represent the urogenital schistosomiasis situation in other regions of Anambra State or Nigeria due to the focal nature of the disease. Secondly, the exclusion of senior secondary class 3 (SS3) students due to the ongoing West African Senior Secondary Examinations (WASSCE) could have resulted in underestimating the prevalence of urogenital schistosomiasis among older adolescents.

For future studies, it is recommended to conduct a cost-effectiveness analysis of integrating FGS diagnosis with sexual and reproductive health services in Nigeria. This analysis could provide valuable insights into the feasibility and potential benefits of such integration, helping policymakers and healthcare providers make informed decisions about resource allocation and design of integrated healthcare programs. Additionally, exploring the impact of this integrated approach on health outcomes and patient satisfaction could further strengthen the evidence base for its implementation.

In conclusion, the findings underscore the importance of revising and expanding current MDA control programs to include secondary school adolescents and other vulnerable groups. This would contribute to better health outcomes and overall well-being in Anambra State.

### Electronic supplementary material

Below is the link to the electronic supplementary material.

Supplementary Material 1

## Data Availability

The datasets used and/or analysed during the current study are available from the corresponding author on reasonable request.

## Abbreviations

AIDS: Acquired Immune Deficiency Syndrome
ANOVA: Analysis of Variance
BCC: Behaviour Change Communication
DALYs: Disability-Adjusted Life Years
FGS: Female Genital Schistosomiasis
FMOH: Federal Ministry of Health
GPS: Global Positioning System
HIV: Human Immunodeficiency Virus
HPV: Human Papilloma Virus
KAP: Knowledge Attitude and Practice
LGA: Local Government Area
MAMs: Mass Administration of Medicines
MGS: Male Genital Schistosomiasis
NTDs: Neglected Tropical Diseases
PC: Preventive Chemotherapy
SAC: School-aged Children
SCC: Squamous Cell carcinoma
SDG: Sustainable Development Goals
STI: Sexually Transmitted Infections
UgS: Urogenital Schistosomiasis
WASSCE: West African Senior School Certificate Examinations
WASH: Water, Sanitation and Hygiene
WHO: World Health Organization
